# Validation of a Semi-Quantitative Food-Frequency Questionnaire for Dutch Pregnant Women from the General Population Using the Method or Triads

**DOI:** 10.3390/nu12051341

**Published:** 2020-05-08

**Authors:** Trudy Voortman, Régine P.M. Steegers-Theunissen, Nienke E. Bergen, Vincent W. V. Jaddoe, Caspar W. N. Looman, Jessica C. Kiefte-de Jong, Sarah Schalekamp-Timmermans

**Affiliations:** 1 Department of Epidemiology, Erasmus MC, 3000 CA Rotterdam, The Netherlands; trudy.voortman@erasmusmc.nl (T.V.); j.c.kiefte@lumc.nl (J.C.K.-d.J.); 2 Department of Obstetrics and Gynecology, 3000 CA Erasmus MC, Rotterdam, The Netherlands; r.steegers@erasmusmc.nl (R.P.S.-T.); nienkebergen@gmail.com (N.E.B.); 3 The Generation R Study Group, Erasmus MC, 3000 CA Rotterdam, The Netherlands; v.jaddoe@erasmusmc.nl; 4 Department of Pediatrics, Erasmus MC, 3000 CA Rotterdam, The Netherlands; 5 Department of Public Health, Erasmus MC, 3000 CA Rotterdam, The Netherlands; c.looman@erasmusmc.nl; 6 Department of Public Health and Primary Care, LUMC Campus, 2511 VA The Hague, The Netherlands

**Keywords:** validation, food-frequency questionnaire, method of triads, cohort, pregnancy

## Abstract

Objective: We aimed to validate a food-frequency questionnaire (FFQ) for Dutch pregnant women, against three 24 h-recalls and blood concentrations of B-vitamins and fatty acids, using the method of triads. Methods: We included 83 pregnant women from the general population of Rotterdam, the Netherlands, at a median gestational age of 15.6 weeks. Participants completed three non-consecutive 24 h-recalls, and subsequently filled out the 293-item FFQ. Participants provided blood samples from which we analyzed serum folate and vitamin B12, as well as red blood cell folate, linoleic acid, and total saturated, monounsaturated, and polyunsaturated fatty acids. Results: Estimated energy intake did not differ between the FFQ and 24 h-recalls. Deattenuated Pearson’s correlation coefficients, between energy-adjusted nutrient intake estimates from the FFQ and the 24 h-recalls, ranged from 0.41 (fat) to 0.88 (fiber) for macronutrients, and were around 0.6 for most micronutrients, except for vitamin E (0.27). Using the triad method, we obtained validity coefficients of 0.86 (95% Confidence Interval (CI) 0.36, 1.00) for serum folate, 0.86 (95% CI 0.18, 1.00) for red blood cell folate, and 1.00 (95% CI 0.42, 1.00) for vitamin B12. Validity coefficients for serum fatty acids ranged from 0.22 to 0.67. Conclusion: This FFQ is a reliable tool for estimating intake of energy, macronutrients, folate and vitamin B12 among women in mid-pregnancy.

## 1. Introduction

Compelling evidence demonstrates a link between diet in pregnancy and birth outcomes, extending to the long-term health of the offspring. During pregnancy, the need for energy and nutrient intake changes in order to meet with the demands of the developing fetus [1,2,3]. The developmental origins of health and disease (DOHaD) model proposes that differences in prenatal exposures such as maternal diet, acting at different stages of fetal development, can cause permanent developmental adaptations thereby not only affecting fetal growth but also the risk of diseases in later life [1]. Diet during pregnancy has indeed been linked to fetal growth [2,3,4,5], later child health [6,7,8], and adult chronic disease risk [9]. Maternal diet has also been associated with various congenital malformations, such as congenital heart defects, cleft defects in palate and lip, and neural tube defects [10,11]. Further, maternal health can be affected by diet during pregnancy, resulting in changes in blood pressure, gestational diabetes and gestational weight gain [12,13,14,15]. Most of this evidence comes from birth cohort studies with prenatal enrolment. These studies require accurate methods to estimate dietary intake during pregnancy.

A food-frequency questionnaire (FFQ) is a self-report method that is often used in large-scale epidemiological studies, and is considered an adequate method of estimating habitual dietary intake. FFQs need to be developed for, and validated in, the specific population of interest, because dietary habits might vary per study population [16]. As no gold standard measurement for overall dietary intake is available, validation of dietary intake questionnaires such as FFQs often consists of comparison with other self-report methods as reference methods, such as 24 h-recalls or food records. With this approach, however, both the FFQ and the reference method are subject to similar measurement errors, which may results in an overestimation of the validity of the FFQ [17]. To overcome this problem of correlated errors, it has been proposed to use biochemical markers of dietary intake as a reference method, of which the errors are likely to be independent of self-reported dietary intake [18,19]. However, most nutritional biomarkers do not reflect only dietary intake. Concentrations of nutrients in tissues also depend on the absorption, metabolism, storage and excretion of these nutrients, causing variation in the biomarker not related to dietary intake. Verkleij-Hagoort et al. described and applied the method of triads as an approach to validate FFQs [19]. With this approach, a triangular comparison between the FFQ, another self-report method, and a nutritional biomarker is made, which allows estimation of validity coefficients adjusted for correlated errors [19]. 

The aim of the current study was to evaluate an FFQ, which was adapted for Dutch pregnant women to primarily estimate B-vitamin intake, using the method of triads, using data from 24 h-recalls and blood concentrations of folate, vitamin B12 and fatty acids. Secondly, we also evaluated intake of other macronutrients and micronutrients against 24 h-recalls.

## 2. Materials and Methods

### 2.1. Study Design and Participants

**Figure 1:**
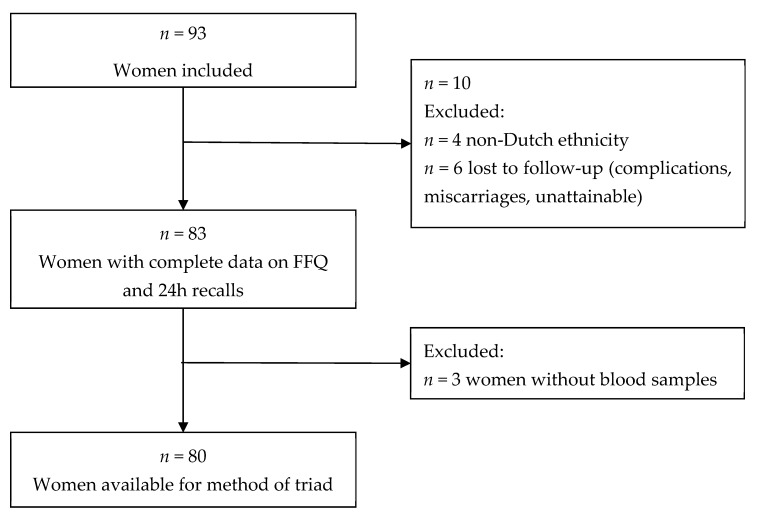
Flow-chart.

We invited Dutch pregnant women between 8 and 24 weeks of gestation from a Dutch urban population (city of Rotterdam, the Netherlands), who attended a midwife as part of their routine pregnancy check-up policy, to participate in the study. Between March 2010 and August 2010, 93 women were included in the study. Of those 93 women, we excluded women with a non-Dutch ethnic background (*n* = 4) and those who were lost to follow-up (*n* = 6), resulting in a population for analysis of 83 women, of whom 80 (96%) had blood samples available (Figure 1). A questionnaire was administered at enrollment in the study to obtain information on participant’s age, due date, pre-pregnancy weight and height, ethnicity, educational level, parity, smoking, whether any dietary restrictions were followed, and vitamin supplement use (folic acid and multivitamin). This information was validated at the first 24 h-recall. The mean (± SD) age of the women was 31.8 years (±4.0), and they had a median gestational age of 15.6 weeks (90% range 8.4–20.4) at enrolment in the study. Most of the women had a high educational level (90.4%), did not smoke (86.7%) and used folic acid supplementation (97.6%) (Table 1).

The study was approved by the Medical Ethical Committee of Erasmus MC, University Medical Center, Rotterdam, the Netherlands (MEC 2007–413). Written informed consent was obtained from all participants. The data are available upon request.

### 2.2. Food-Frequency Questionnaire

The applied FFQ is a self-administered 293-item questionnaire, in which participants reported their food intake during the previous three months. This FFQ has been applied in the Generation R Study, a population-based cohort study from fetal life onwards in Rotterdam, the Netherlands [21]. It is a modified and extended version of a FFQ that was previously developed for and validated in Dutch middle-aged and elderly people [22], and was adapted for use in pregnant women to primarily estimate B-vitamin intake. The FFQ is structured by meal patterns and includes questions on consumption frequency of food items, portion sizes, preparation methods and additions to the dish (Supplemental Table S1). Portion sizes are assessed in standard household measures and using photographs showing different portion sizes (Supplemental Figures S1 and S2) [23]. Participants for this validation study filled out the FFQ once, three weeks after a completed series of three 24 h-recalls. The FFQ covered food intake during the previous three months, thereby covering the three months period over which the participants reported their dietary intake in the FFQ. Average daily energy and nutrient intakes from food intake were calculated using the 2006 Dutch Food Composition Table (NEVO).

### 2.3. 24 Hour-recalls

Three non-consecutive 24 h-recalls were performed before filling out the FFQ. Participants were contacted for the first 24 h-recall within one week after inclusion in the study (median 16.6 weeks (90% range 9.7–21.4)). Researchers who performed the 24 h-recalls all received a training program by a research dietician of Wageningen University. The standardized telephone interview took on average 20 min. Women reported their dietary intake of the previous 24 h, starting from breakfast of the day before. Regarding the estimation of portion sizes, similar photographs as in the FFQ were used (Supplemental Figure S1). In total, three dietary 24 h-recalls were performed over a period of three weeks; one 24 h recall per week (interval between first and second recall: median 1.3 weeks (90% range 0.6–2.4); interval between second and third recall: median 1.1 weeks (90% range 0.4–2.4))**.** The FFQ was sent to the participants three weeks after the third 24 h-recall. We aimed to conduct the 24 h-recalls covering two weekdays and one weekend day. Median food intake of the three 24 h-recalls were used to calculate nutrient intakes. These were calculated on the basis of the 2006 Dutch Food Composition Table (NEVO) using Komeet software (Komeet 3.0, BaS Nutrition Software, Wageningen, the Netherlands) [24]. 

Nutrient intakes obtained from the FFQ and 24 h-recalls were adjusted for total energy intake using the nutrient residual method [25]. Basal metabolic rate (BMR) was calculated using the revised Harris–Benedict equation [20]. 

### 2.4. Biomarkers

After the last 24 h-recall, an appointment was made to collect venous blood samples for the measurement of nutritional biomarkers (median 19.8 weeks (90% range 13.4–23.9)). Blood was collected in an 8.5 mL Vacutainer Serum Separator Tube for the determination of serum folate and vitamin B12 concentrations, and in a 10 mL Vacutainer ethylenediaminetetraacetic acid (EDTA) tube (BD Diagnostics, Plymouth, UK) to determine red blood cell (RBC) folate and fatty acid concentrations. To account for potential influences of recent dietary intake, we chose to assess serum folate and vitamin B12 concentrations as well as RBC folate concentrations, thereby reflecting both short- and long-term folate status.

At the Department of Clinical Chemistry at Erasmus MC Rotterdam, serum folate and vitamin B12 concentrations, and RBC folate concentrations, were analyzed using an immunoelectrochemoluminence assay on the Architect System (Abbott Diagnostics B.V., Hoofddorp, the Netherlands). For analysis of fatty acid concentrations, EDTA plasma samples were stored at minus –80 °C and transported to the Division of Metabolic Diseases and Nutritional Medicine, Dr. von Hauner Children’s Hospital, Ludwig-Maximilians University of Munich, in 2012. After being thawed, the analysis of plasma glycerophospholipid fatty acid composition was performed by a sensitive and precise high-throughput method [26]. The average coefficient of variation was 3.2%. For the current analyses, we used concentrations of linoleic acid (C18:2 *n*-6), the sum of saturated fatty acids (SFA), the sum of monounsaturated fatty acids (MUFA), and the sum of polyunsaturated fatty acids (PUFA) in the chromatogram.

### 2.5. Statistical Analysis

Estimates from the FFQ were evaluated against those from the 24 h-recalls using three approaches. First, average daily energy intake and intake of nutrients as estimated from the FFQ and 24 h-recalls were compared using paired sample *t*-tests. Second, Pearson’s correlation coefficients were calculated to evaluate the linear correlations between the estimates obtained with the FFQ and the 24 h-recalls. To correct for day-to-day variation in the 24 h-recalls, we de-attenuated the crude correlation coefficients by multiplying them with a factor (1+(σ_intra_^2^/σ_inter_^2^)/*n*)^1/2^, where *n* is the number of repeated 24 h-recalls per participant, σ_intra_^2^ is the intra-individual variance, and σ_inter_^2^ is the inter-individual variance between the 24 h-recalls [27]. Third, intra-class correlation coefficients (ICC) were computed to evaluate agreement in energy and nutrient intake classification, as assessed with the FFQ and with the 24 h-recalls (< 0.5 = poor agreement, 0.5–0.8 = average agreement, > 0.8 = good agreement). For nutrient intakes with an ICC < 0.5, the presence of any systematic difference between the two methods was evaluated with a Bland–Altman Plot, and by calculating the limits of agreement as the mean difference ±2 SD.

Estimated intakes of folate, vitamin B12 and fatty acids from the FFQ were additionally evaluated against estimates from both the 24 h-recalls and biomarkers of these nutrients, using the method of triads. This approach assumes that correlations between the three measurements are explained completely by their linear relations to the unknown ‘true’ intake, and that their random errors are independent of each other [17,18]. Confidence intervals for the validity coefficients were estimated using bootstrap sampling, where 1000 samples of equal size (*n* = 80) were obtained by random sampling with replacement [17]. As blood concentrations of nutrients can be affected by factors other than diet, we performed sensitivity analyses to adjust the method of triads for Body mass index (BMI), smoking, and gestational age at blood sampling time point.

Analyses were performed using Statistical Package for the Social Sciences (SPSS) for Windows version 21.0 (IBM Corp, Armonk, NY, USA) and R version 3.1.2 (R Foundation for Statistical Computing, Vienna, Austria).

## 3. Results

### 3.1. Evaluation of the FFQ Against 24 h-Recalls

Energy and nutrient intakes estimated by FFQ and the repeated 24 h-recalls are presented in Table 2. Estimated intake of energy and protein was not statistically significantly different between the two methods. Intake of total fat, particularly of PUFA, was slightly overestimated by the FFQ compared to the 24 h-recalls, whereas intake of carbohydrates, particularly polysaccharides, was slightly underestimated. These results were similar for nutrient intakes adjusted for energy intake (Supplemental Table S2). After correction for day-to-day variation of the repeated 24 h-recalls, the correlation coefficient was 0.30 for total energy intake, and the correlations ranged from 0.38 (MUFA) to 0.88 (fiber) for intake of macronutrients. For intake of micronutrients, deattenuated correlation coefficients were 0.6 or higher, except for intake of vitamin E (r = 0.27) and vitamin B12 (r = 0.46) (Table 2). ICCs were > 0.5 for most of the nutrients, except for estimated intake of MUFAs, retinol, vitamin B1, vitamin B12 and vitamin E (Table 2). Bland–Altman plots showed no systematic difference between both methods for estimation of energy-adjusted MUFA or vitamin E intake. However, for retinol, vitamin B1 and vitamin B12, the FFQ appeared to underestimate intake compared to the 24 h-recalls for the subjects with the highest intakes (Supplemental Figures S3–S7).

### 3.2. Evaluation of the FFQ Against 24 h-recalls and Biomarkers: the Method of Triads

The results from the method of triads are presented in Table 3. The correlations of folate intake estimated with the FFQ and the biomarkers (lower limits of the validity coefficients) were 0.24 (95% CI −0.01, 0.47) for serum folate, and 0.09 (95% CI −0.19, 0.37) for RBC folate, and the estimated validity coefficient obtained with the triad method (upper limit) was 0.86 (95% CI 0.36, 1.00) for serum folate, and 0.86 (95% CI 0.18, 1.00) for RBC folate.

For estimated vitamin B12 intake, the correlation with the serum vitamin B12 was 0.33 (95% CI 0.08, 0.54) and the estimated validity coefficient of the method of triads was even higher than 1 (1.10), and was set to 1 (1.00 (95% CI 0.42, 1.00)). For the fatty acids, however, correlations between serum levels and estimated intakes were low (−0.17 to 0.16), and validity coefficients with the method of triads were 0.22 (95% CI 0.12, 1.00) for SFAs, 0.58 (95% CI 0.13, 1.00) for MUFAs, 0.33 (95% CI 0.13, 1.00) for PUFAs, and 0.67 (95% CI 0.20, 1.00) for linoleic acid (Table 3). Additional adjustment for maternal BMI, gestational age at blood sampling and maternal smoking improved the coefficients for SFAs (validity coefficient 0.64 (95% CI 0.19, 1.00)), but had no large effect on the estimates for the other nutrients (Supplemental Table S2).

## 4. Discussion

We evaluated the validity of an FFQ for Dutch pregnant women against multiple 24 h-recalls, and against a combination of 24 h-recalls and blood levels of folate, vitamin B12 and fatty acids, using the method of triads [19]. In the evaluation against the 24 h-recalls, we observed moderate to good validity for intake of energy and macronutrients. Using the method of triads, we observed good validity for intake of folate and vitamin B12, but low to moderate validity for estimation of intake of fatty acids.

In the current study, we evaluated a FFQ for pregnant women using two approaches. First, we compared estimates obtained by the FFQ with those obtained via multiple 24 h-recalls. From this evaluation, we observed that estimated energy intake did not differ between these two methods, and that the validity for estimation of intake of most nutrients was moderate to good. However, FFQs and 24 h-recalls both rely on self-report and are thereby subject to similar measurement errors. Positively correlated measurement errors may lead to an overestimation of the validity of the FFQ [17]. 

To improve estimates of the validity of dietary assessment, biochemical markers of dietary intake can be included as a reference method, of which the errors are likely independent of self-reported dietary intake [18,19]. The method of triads estimates the validity of a dietary assessment method by comparing it with both another self-report reference method and nutritional biomarkers. This approach is based on the assumption that all three measurements have a linear relation with the unknown true intake, and that random errors between the three methods are independent of each other [18]. As the latter assumption may not be true for the 24 h-recalls and the FFQ, which are not expected to have fully independent errors, we interpreted the coefficient from the method of triads as the upper limit of the true validity coefficient. And, as correlations between biomarkers are affected by several other factors than dietary intake, we interpreted the correlation between the FFQ and the single biomarker measurement as the lower limit of the validity coefficient. With this approach we observed, for example, for serum folate, a lower limit of the validity coefficient of 0.24, and an upper limit of 0.86. For vitamin B12, the originally observed validity coefficient from the method of triads was even higher than 1. This occurs if the product of two of the correlations between the dietary methods is much greater than the third correlation, and is known as a Heywood case [17]. In our study, the correlation between serum vitamin B12 and vitamin B12 intake estimated with the 24 h-recalls was much lower than the other two correlations in the triad. Both serum vitamin B12 and RBC folate reflect long term parameters. Other explanations could be the random sampling variation in the correlations between methods, but also violations in the model assumptions of the triad method [17]. 

Validity coefficients were generally low for the different fatty acid subgroups [28]. This is in line with several other studies that also showed poor correlations between dietary fatty acid intake and blood levels, varying from correlation coefficients between −0.02 and 0.83 for alpha-linolenic acid, 0.35 and 0.61 for docosahexaenoic acid, and 0.36 and 0.91 for eicosapentaenoic acid. Blood levels of fatty acids may not be good proxies for intake of fatty acids [29,30,31]. Serum levels of most fatty acids are supplied not only by dietary fat intake, but also by endogenous synthesis. Only a few fatty acids can be considered as adequate markers of dietary intake, such as the PUFAs alpha-linolenic and linoleic acid, which cannot be synthesized de novo [32]. In line with this, observed validity coefficients were higher for linoleic acid than for total saturated, monounsaturated or polyunsaturated fatty acids, suggesting that the FFQ may not be necessarily poor in estimating fatty acid intake, but that rather the biomarkers may not be valid for estimating dietary intake. Furthermore, we used plasma phospholipid fatty acids, reflecting only dietary intake in the relatively short term [32]. 

In general, although biomarkers can be measured more objectively, they do not necessarily reflect dietary intake better than self-reported intake. Only a few biomarkers (i.e., recovery biomarkers) directly reflect quantitative dietary intake, e.g., 24 h urinary nitrogen for protein intake. However, most nutritional biomarkers, including those in our study, are concentration markers which are expected to be correlated with intake, but do not reflect absolute intake of nutrient intakes. Concentrations of these biomarkers are often only weakly correlated with dietary intake, with values < 0.4 [33]. The remaining variation in biomarker concentrations can be explained by differences in absorption, tissue uptake, metabolism and excretion of these nutrients, due to, for example, genetic differences in Fatty acid desaturase (FADS2) or Methylene tetrahydrofolate reductase (MTHFR) genes, environmental factors such as smoking and weight gain, or specific pregnancy-related changes that might also influence nutrient concentrations [19,34]. However, in contrast to results from previous studies in general populations, adjustment for smoking, BMI, or gestational age did not affect estimates for the correlations with intake in our analyses [35]. 

The strengths of this study are the recruitment and inclusion of participants from a general pregnant population, and the estimation of both short- and long-term biomarkers. However, our study was restricted to women living in the city of Rotterdam, and the majority of women was highly educated, thereby not completely reflecting the general Dutch pregnant population. Furthermore, we selected only those with a Dutch ethnic background because the FFQ was developed for a Dutch diet. We also did not have detailed data on early pregnancy complications or pre-existing diseases of the study participants. Larger validation studies in other subgroups would be needed to verify the adequacy of this FFQ for women with other ethnic or socioeconomic backgrounds. The 24 h-recall estimates dietary intake on a single day, and may therefore not accurately represent habitual diet. For this reason, three recalls were performed, distributed over a period of three weeks within the reference period of the FFQ. More frequent 24 h-recalls would have further improved our estimates. However, we took intra-individual variance into account, which showed average−good agreement. Overall, we observed reasonable to good validity for most nutrients, as estimated with the FFQ. Generally, and in line with previous studies, validity estimates improved after adjusting for energy intake, underscoring the importance of adjusting nutrient intakes for total energy intake, not only to control for confounding by energy intake but also to improve estimations of intake [36]. Although validity was not perfect, the FFQ can be used to rank women according to their nutrient intakes and study the associations of this with health, acknowledging the validity of the nutrient intake estimations in interpreting the results. In the meantime, continued effort is needed to further improve dietary intake estimation in large-scale epidemiological studies, e.g., by combining data obtained from both long-term instruments, such as FFQs, and short-term instruments, such as 24 h-recalls or food records, also making use of web-based tools like biomarkers [36,37]. 

## 5. Conclusions

In this validation study applying the method of triads, we observed that this 293-item FFQ is a reliable tool for estimating intake of energy, macronutrients, folate and vitamin B12 among pregnant women, but it may need to be adapted for more accurate assessment of intake of fatty acid subgroups. Research using data obtained with this FFQ should acknowledge the validity of the estimation in interpreting associations with health outcomes. Further research remains needed, to develop accurate and cost-effective tools for estimating individual usual dietary intake in large-scale epidemiological studies.

## Figures and Tables

**Table 1:** Participant characteristics.

Variable (Unit)	Value *
Age (years)	31.8 *±* 4.0
Gestational age at enrollment (weeks)	15.6 (8.4–20.4)
Weight (kg)	66.0 (53.2–87.2)
Height (m)	1.72 *±* 0.1
Body mass index (kg/m^2^)	22.0 (18.4–29.7)
Basal metabolic rate (kcal) [20]	1756 (1632–1916)
Educational level	
*Low*	1 (1.2%)
*Medium*	7 (8.4%)
*High*	75 (90.4%)
Vegetarian diet	
*No*	78 (94.0%)
*No meat and no fish*	2 (2.4%)
*No meat*	3 (3.6%)
Parity	
*Nulliparous*	50 (60.2%)
*Multiparous*	33 (39.8%)
Smoking	
*No*	72 (86.7%)
*Yes, until pregnancy was known*	7 (8.4%)
*Yes, continued*	4 (4.8%)
Folic acid supplement use	
*No*	2 (2.4%)
*When pregnancy was known*	24 (28.9%)
*Later in pregnancy*	3 (3.6%)
*Preconceptionally*	54 (65.1)
Biomarker concentrations at median 19.8 weeks (90% range 13.4–23.9) of gestation	
Serum folate (nmol/L)	30.6 *±* 8.9
Red blood cell folate (nmol/L)	1479.7 *±* 301.5
Serum vitamin B_12_ (pmol/L)	247.0 *±* 74.1
Plasma saturated fatty acids (mg/L)	688.6 *±* 117.5
Plasma monounsaturated fatty acids (mg/L)	217.3 *±* 37.3
Plasma polyunsaturated fatty acids (mg/L)	709.4 *±* 113.9
Plasma linoleic acid (mg/L)	349.0 *±* 59.4

* Values are mean ± SD for continuous variable with a normal distribution, median (90% range) for continuous variables with a skewed distribution, or number (percentage) for categorical variables.

**Table 2:** Energy and nutrient intakes as estimated with the FFQ and repeated 24 h-recalls.

	Estimated Nutrient Intakes (Median (90% Range))			Pearson’s Correlation Coefficients			Intraclass Correlation Coefficients	
	FFQ	24 h-Recalls	*p*- Value	Crude	Energy- Adjusted ^†^	De-Attenuated ^‡^	Crude	Energy Adjusted
Energy (kcal/d)	2099 (1344–2995)	2123 (1528–2830)	0.76	0.23 *	-	0.30	0.36 *	-
Total protein (g/d)	74.7 (48.3–109.0)	77.9 (56.0–105.6)	0.31	0.25 *	0.46 **	0.39	0.39 **	0.62 **
Vegetable protein (g/d)	32.3 (20.5–53.1)	32.7 (19.7–48.0)	0.43	0.52 **	0.65 **	0.74	0.62 **	0.79 **
Animal protein (g/d)	43.7 (23.4–62.9)	44.9 (24.4–65.7)	0.15	0.27 *	0.46 **	0.46	0.42 **	0.62 **
Total fat (g/d) **^§^**	87.1 (53.0–140.2)	80.1 (49.9–120.1)	0.05	0.28 *	0.42 **	0.41	0.43 **	0.58 **
SFA (g/d) ^**§**^	28.4 (14.9–48.9)	31.0 (17.0–48.6)	0.19	0.40 **	0.60 **	0.60	0.57 **	0.75 **
MUFA (g/d) ^**§**^	30.2 (17.2–49.2)	30.0 (16.0–45.4)	0.02	0.25 *	0.33 **	0.38	0.40 *	0.47 **
PUFA (g/d) ^**§**^	19.5 (10.6–33.7)	14.2 (7.9–22.6)	<0.001	0.42 **	0.54 **	0.69	0.59 **	0.69 **
Total carbohydrates (g/d)	248.3 (148.2–358.0)	266.7 (183.2–398.4)	0.02	0.32 **	0.43 **	0.42	0.49 **	0.60 **
Mono- and disaccharides (g/d) |	134.6 (72.2–213.7)	128.8 (81.1–253.4)	0.75	0.44 **	0.58 **	0.50	0.61 **	0.74 **
Polysaccharides (g/d)	112.1 (68.7–165.5)	131.8 (86.3–193.3)	<0.001	0.33 **	0.41 **	0.55	0.5 **	0.58 **
Fiber (g/d)	24.5 (12.5–42.4)	23.8 (13.4–37.3)	0.66	0.63 **	0.65 **	0.88	0.77 **	0.77 **
Calcium (mg/d)	1145 (516–2035)	1189 (779–1657)	0.71	0.45 **	0.60 **	0.68	0.59 **	0.74 **
Phosphor (g/d)	1535 (894–2285)	1531 (1080–2200)	0.78	0.42 **	0.62 **	0.60	0.58 **	0.76 **
Total iron (mg/d)	12.2 (7.5–18.8)	11.9 (8.1–17.3)	0.93	0.47 **	0.45 **	0.62	0.64 **	0.61 **
Retinol equivalents (mg/d) ^**§**^	877 (380–1351)	671 (293–1844)	0.01	0.37 **	0.17	0.62	0.52 **	0.22
Vitamin B1 (mg/d) ^**§**^	1.2 (0.6–1.9)	1.3 (0.8–2.5)	0.16	0.34 **	0.24 *	0.60	0.5 **	0.35 *
Vitamin B2 (mg/d)	1.5 (0.8–2.5)	1.7 (0.9–2.4)	0.007	0.47 **	0.58 **	0.61	0.64 **	0.72 **
Vitamin B6 (mg/d) **^§^**	1.9 (1.0–3.2)	1.9 (1.1–3.4)	0.40	0.49 **	0.55 **	0.59	0.65 **	0.67 **
Vitamin B12 (µg/d) **^§^**	3.1 (1.7–4.9)	3.3 (1.6–7.0)	0.20	0.24 *	0.24 *	0.46	0.37 *	0.33 *
Folate equivalents (µg/d) **^§^**	178 (117–305)	213 (114–402)	0.001	0.43 **	0.58 **	0.62	0.59 **	0.67 **
Vitamin E (mg/d)	17.2 (9.0–28.8)	14.2 (6.8–21.9)	<0.001	0.18	0.23 *	0.27	0.29	0.37 *
Vitamin C (mg/d) |	101.8 (46.6–215.1)	120.7 (41.6–260.1)	0.01	0.52 **	0.44 **	0.65	0.68 **	0.59 **

Abbreviations: FFQ, food-frequency questionnaire; SFA, the sum of saturated fatty acids; MUFA, the sum of monounsaturated fatty acids; PUFA, the sum of polyunsaturated fatty acids (PUFA). Comparison of the estimates of the FFQ and 24 h-recall was performed with paired *t*-tests † Adjusted for energy using the residual method (Willett et al., 1997) ‡ Corrected for day-to-day variation of the repeated 24 h-recalls (Rosner and Willett, 1988) § Log-transformed for analysis, | Square root-transformed for analysis, * *p*-value < 0.05, ** *p*-value < 0.01.

**Table 3:** Correlation coefficients between estimates from each of the three assessment methods, and the validity coefficients calculated with the method of triads.

				Fatty Acids (Plasma Phospholipids)			
	Serum Folate	RBC Folate	Serum Vitamin B_12_	Saturated Fatty Acids	Mono- Unsaturated Fatty Acids	Poly- Unsaturated Fatty Acids	Linoleic Acid
	*n* = 80	*n* = 77	*n* = 80	*n* = 78	*n* = 78	*n* = 78	*n* = 78
Sample correlations							
r_QM_	0.24	0.09	0.33	0.01	−0.17	−0.02	0.16
(95% CI)	(−0.01, 0.47)	(−0.19, 0.37)	(0.08, 0.54)	(−0.19, 0.21)	(−0.36, 0.05)	(−0.21, 019)	(−0.02, 0.33)
r_RM_	0.14	0.05	0.07	0.09	−0.12	−0.08	0.13
(95% CI)	(−0.07, 0.37)	(−0.20, 0.30)	(−0.15, 0.27)	(−0.10, 0.29)	(−0.35, 0.10)	(−0.30, 0.14)	(−0.10, 0.36)
r_QR_	0.43	0.41	0.24	0.41	0.24	0.42	0.36
(95% CI)	(0.22, 0.59)	(0.19, 0.58)	(0.07, 0.41)	(0.18, 0.62)	(−0.01, 0.46)	(0.17, 0.62)	(0.10, 0.59)
Validity coefficient *							
ρ_QT_	0.86	0.86	1.00	0.22	0.58	0.33	0.67
(95% CI)	(0.36, 1.00)	(0.18, 1.00)	(0.42, 1.00)	(0.12, 1.00)	(0.13, 1.00)	(0.13, 1.00)	(0.20, 1.00)
Range **^†^**	0.24–0.86	0.09–0.86	0.33–1.00	0.01–0.22	−0.17–0.58	−0.02–0.33	0.16–0.67

Abbreviations: rQM, correlation between FFQ and biomarker; rRM, correlation between 24 h-recalls and biomarker; rQR, correlation between FFQ and 24 h-recalls; rQT, validity coefficient of the FFQ; 95% CI, 95% confidence interval; RBC, red blood cell; FFQ, food frequency questionnaire. * Validity coefficients and confidence interval limits above 1 were set to 1.00. **†** Range: The lower limit is rQM and the upper limit is ρ_QT_ (Ocke and Kaaks, 1997).

## Supplementary Materials

The following are available online at https://www.mdpi.com/2072-6643/12/5/1341/s1, Figure S1: Photographs with portion sizes, Figure S2: Food frequency questionnaire, Figures S3–S7: Bland-Altman plots, Table S1: Food groups included in the FFQ, Table S2: Correlation coefficients between each of the three assessment methods and the validity coefficient calculated with the method of triads, adjusted for covariates.

## References

[1] Gillman M.W. (2005). Developmental origins of health and disease. N. Engl. J. Med..

[2] Bouwland-Both M.I., Steegers-Theunissen R.P., Vujkovic M., Lesaffre E.M., Mook-Kanamori D.O., Hofman A., Lindemans J., Russcher H., Jaddoe V.W., Steegers E.A. (2013). A periconceptional energy-rich dietary pattern is associated with early fetal growth: The generation r study. BJOG.

[3] Parisi F., Rousian M., Steegers-Theunissen R.P.M., Koning A.H.J., Willemsen S.P., de Vries J.H.M., Cetin I., Steegers E.A.P. (2018). Early first trimester maternal ‘high fish and olive oil and low meat’ dietary pattern is associated with accelerated human embryonic development. Eur. J. Clin. Nutr..

[4] Timmermans S., Steegers-Theunissen R.P., Vujkovic M., den Breeijen H., Russcher H., Lindemans J., Mackenbach J., Hofman A., Lesaffre E.E., Jaddoe V.V. (2012). The mediterranean diet and fetal size parameters: The generation r study. Br. J. Nutr..

[5] Oostingh E.C., Hall J., Koster M.P.H., Grace B., Jauniaux E., Steegers-Theunissen R.P.M. (2019). The impact of maternal lifestyle factors on periconception outcomes: A systematic review of observational studies. Reprod. Biomed. Online.

[6] Jen V., Erler N.S., Tielemans M.J., Braun K.V., Jaddoe V.W., Franco O.H., Voortman T. (2017). Mothers’ intake of sugar-containing beverages during pregnancy and body composition of their children during childhood: The generation r study. Am. J. Clin. Nutr..

[7] Steenweg-de Graaff J., Tiemeier H., Steegers-Theunissen R.P., Hofman A., Jaddoe V.W., Verhulst F.C., Roza S.J. (2014). Maternal dietary patterns during pregnancy and child internalising and externalising problems. The generation r study. Clin. Nutr..

[8] Shaheen S.O., Northstone K., Newson R.B., Emmett P.M., Sherriff A., Henderson A.J. (2009). Dietary patterns in pregnancy and respiratory and atopic outcomes in childhood. Thorax.

[9] de Rooij S.R., Painter R.C., Holleman F., Bossuyt P.M., Roseboom T.J. (2007). The metabolic syndrome in adults prenatally exposed to the dutch famine. Am. J. Clin. Nutr..

[10] Vujkovic M., Steegers E.A., Looman C.W., Ocke M.C., van der Spek P.J., Steegers-Theunissen R.P. (2009). The maternal mediterranean dietary pattern is associated with a reduced risk of spina bifida in the offspring. BJOG.

[11] Obermann-Borst S.A., Vujkovic M., de Vries J.H., Wildhagen M.F., Looman C.W., de Jonge R., Steegers E.A., Steegers-Theunissen R.P. (2011). A maternal dietary pattern characterised by fish and seafood in association with the risk of congenital heart defects in the offspring. BJOG.

[12] Han S., Crowther C.A., Middleton P., Heatley E. (2013). Different types of dietary advice for women with gestational diabetes mellitus. Cochrane Database Syst. Rev..

[13] Tielemans M.J., Garcia A.H., Peralta Santos A., Bramer W.M., Luksa N., Luvizotto M.J., Moreira E., Topi G., de Jonge E.A., Visser T.L. (2016). Macronutrient composition and gestational weight gain: A systematic review. Am. J. Clin. Nutr..

[14] Timmermans S., Steegers-Theunissen R.P., Vujkovic M., Bakker R., den Breeijen H., Raat H., Russcher H., Lindemans J., Hofman A., Jaddoe V.W. (2011). Major dietary patterns and blood pressure patterns during pregnancy: The generation r study. Am. J. Obstet. Gynecol..

[15] Hillesund E.R., Overby N.C., Engel S.M., Klungsoyr K., Harmon Q.E., Haugen M., Bere E. (2014). Associations of adherence to the new nordic diet with risk of preeclampsia and preterm delivery in the norwegian mother and child cohort study (moba). Eur. J. Epidemiol..

[16] Willett W. (2013). Nutritional Epidemiology.

[17] Ocke M.C., Kaaks R.J. (1997). Biochemical markers as additional measurements in dietary validity studies: Application of the method of triads with examples from the european prospective investigation into cancer and nutrition. Am. J. Clin. Nutr..

[18] Kaaks R.J. (1997). Biochemical markers as additional measurements in studies of the accuracy of dietary questionnaire measurements: Conceptual issues. Am. J. Clin. Nutr..

[19] Verkleij-Hagoort A.C., de Vries J.H., Stegers M.P., Lindemans J., Ursem N.T., Steegers-Theunissen R.P. (2007). Validation of the assessment of folate and vitamin b12 intake in women of reproductive age: The method of triads. Eur. J. Clin. Nutr..

[20] Roza A.M., Shizgal H.M. (1984). The harris benedict equation reevaluated: Resting energy requirements and the body cell mass. Am. J. Clin. Nutr..

[21] Jaddoe V.W., van Duijn C.M., Franco O.H., van der Heijden A.J., van Iizendoorn M.H., de Jongste J.C., van der Lugt A., Mackenbach J.P., Moll H.A., Raat H. (2012). The generation r study: Design and cohort update 2012. Eur. J. Epidemiol..

[22] Klipstein-Grobusch K., den Breeijen J.H., Goldbohm R.A., Geleijnse J.M., Hofman A., Grobbee D.E., Witteman J.C. (1998). Dietary assessment in the elderly: Validation of a semiquantitative food frequency questionnaire. Eur. J. Clin. Nutr..

[23] Donders-Engelen M., van der Heijden L. (2003). Maten, Gewichten en Codenummers 2003.

[24] Netherlands Nutrition Centre (2006). Dutch Food Composition Database 2006 (Nevo-Table 2006).

[25] Willett W.C., Howe G.R., Kushi L.H. (1997). Adjustment for total energy intake in epidemiologic studies. Am. J. Clin. Nutr..

[26] Steenweg-de Graaff J.C., Tiemeier H., Basten M.G., Rijlaarsdam J., Demmelmair H., Koletzko B., Hofman A., Jaddoe V.W., Verhulst F.C., Roza S.J. (2015). Maternal lc-pufa status during pregnancy and child problem behavior: The generation r study. Pediatr. Res..

[27] Rosner B., Willett W.C. (1988). Interval estimates for correlation coefficients corrected for within-person variation: Implications for study design and hypothesis testing. Am. J. Epidemiol..

[28] McNaughton S.A., Hughes M.C., Marks G.C. (2007). Validation of a ffq to estimate the intake of pufa using plasma phospholipid fatty acids and weighed foods records. Br. J. Nutr..

[29] Swierk M., Williams P.G., Wilcox J., Russell K.G., Meyer B.J. (2011). Validation of an australian electronic food frequency questionnaire to measure polyunsaturated fatty acid intake. Nutrition.

[30] Parra M.S., Schnaas L., Meydani M., Perroni E., Martinez S., Romieu I. (2002). Erythrocyte cell membrane phospholipid levels compared against reported dietary intakes of polyunsaturated fatty acids in pregnant mexican women. Public Health Nutr..

[31] Parker G., McClure G., Hegarty B.D., Smith I.G. (2015). The validity of a food frequency questionnaire as a measure of pufa status in pregnancy. BMC Pregnancy Childbirth.

[32] Arab L. (2003). Biomarkers of fat and fatty acid intake. J. Nutr..

[33] van ’t Veer P., Kardinaal A.F., Bausch-Goldbohm R.A., Kok F.J. (1993). Biomarkers for validation. Eur. J. Clin. Nutr..

[34] Cade J., Thompson R., Burley V., Warm D. (2002). Development, validation and utilisation of food-frequency questionnaires—A review. Public Health Nutr..

[35] Freedman L.S., Commins J.M., Moler J.E., Arab L., Baer D.J., Kipnis V., Midthune D., Moshfegh A.J., Neuhouser M.L., Prentice R.L. (2014). Pooled results from 5 validation studies of dietary self-report instruments using recovery biomarkers for energy and protein intake. Am. J. Epidemiol..

[36] Subar A.F., Freedman L.S., Tooze J.A., Kirkpatrick S.I., Boushey C., Neuhouser M.L., Thompson F.E., Potischman N., Guenther P.M., Tarasuk V. (2015). Addressing current criticism regarding the value of self-report dietary data. J. Nutr..

[37] Conrad J., Nothlings U. (2017). Innovative approaches to estimate individual usual dietary intake in large-scale epidemiological studies. Proc. Nutr. Soc..

