# Supplementary material for: Validation of a Semi-Quantitative Food-Frequency Questionnaire for Dutch Pregnant Women from the General Population Using the Method or Triads
Source: Nutrients. 2020 May 8;12(5):1341. doi: 10.3390/nu12051341 (PMC7284899; doi:10.3390/nu12051341)
Supplement: Supplementary file 1 [file nutrients-12-01341-s001.zip › Supplemental Figure S1 (portion sizes).pdf]

Supplemental Figure S1

Photographs showing different portion sizes

A6. **Hoeveel aardappelen** eet u bij een gemiddelde warme maaltijd?

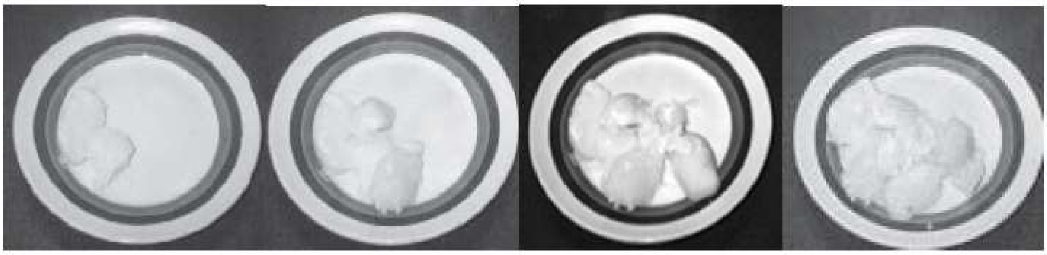

a. ❑ b. ❑ c. ❑ d. ❑

❑ Ik eet geen aardappelen

A7. **Hoeveel** eet u gemiddeld van een **eenpansgerecht of stoofschotel**?

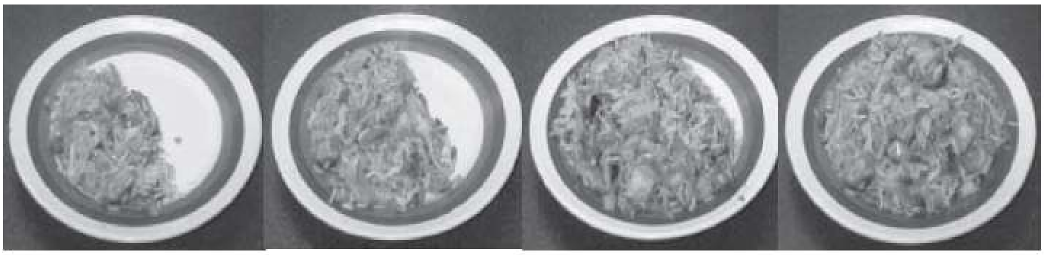

a. ❑ b. ❑ c. ❑ d. ❑

A8. **Hoeveel rijst** eet u bij een gemiddelde warme maaltijd?

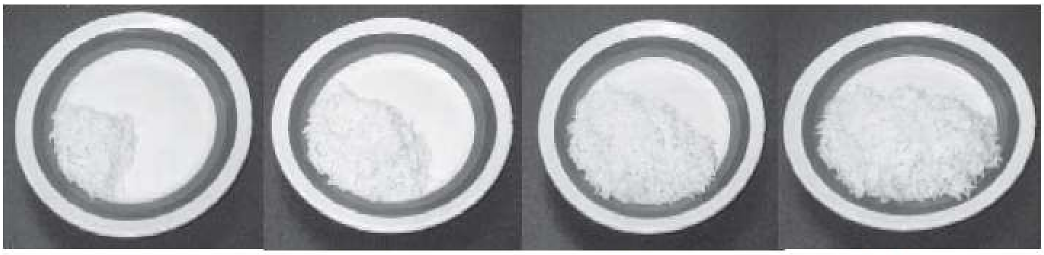

a. ❑ b. ❑ c. ❑ d. ❑

❑ Ik eet geen rijst
