# Supplementary material for: Validation of a Semi-Quantitative Food-Frequency Questionnaire for Dutch Pregnant Women from the General Population Using the Method or Triads
Source: Nutrients. 2020 May 8;12(5):1341. doi: 10.3390/nu12051341 (PMC7284899; doi:10.3390/nu12051341)
Supplement: Supplementary file 1 [file nutrients-12-01341-s001.zip › Supplemental Figure S2 (FFQ).pdf]

F-12345678

VRAGENLIJST THEMA: DE VOEDING VAN MOEDER (Nederlands voedingspatroon)

Dit is de tweede vragenlijst van Generation R. Deze vragenlijst gaat over de voeding van moeder in de **afgelopen 3 maanden**.

Uw antwoorden helpen ons te begrijpen hoe de voeding van de aanstaande moeder de groei en ontwikkeling van het kind voor en na de geboorte beïnvloedt. Het invullen van deze vragenlijst duurt ongeveer 25 minuten.

De gegevens uit deze vragenlijsten worden vertrouwelijk behandeld en gecodeerd verwerkt. Uw naam en adres zijn niet opgenomen in de vragenlijst. We zouden het erg op prijs stellen als u zoveel mogelijk vragen zou beantwoorden, maar we hebben er begrip voor als er vragen zijn die u niet wilt of kunt beantwoorden.

Op de volgende bladzijde volgt een **instructie** voor het invullen van deze lijst. Mocht u problemen hebben bij het beantwoorden van de vragen, dan kunt u ons bellen op het telefoonnummer **010-4087405**. Na het invullen kunt u de vragenlijst in de meegeleverde envelop naar ons toe sturen. Een postzegel is niet nodig.

INHOUDSOPGAVE

A. WARME GERECHTEN ... 3
B. GROENTEN ... 7
C. VLEES, VIS EN VEGETARISCHE PRODUCTEN ... 10
D. BROOD EN BELEG ... 15
E. ZUIVELPRODUCTEN ... 18
F. FRUIT ... 20
G. DRANKEN ... 21
H. TUSSENDOORTJES ... 24
I. OVERIG ... 26

INSTRUCTIE VOOR HET INVULLEN VAN DE VRAGEN

De lijst wordt zoveel mogelijk electronisch verwerkt. Daarom willen wij u vragen deze lijst volgens de onderstaande instructie in te vullen met een **zwarte of blauwe pen**. De vragen in deze lijst gaan over uw voeding in **de afgelopen 3 maanden.**
Soms wordt er naar een merk en/of een verpakking gevraagd. Dit wordt gedaan om de juiste samenstelling van een product te achterhalen.

Meerkeuzevragen

Het is de bedoeling dat u, tenzij anders vermeld, steeds **één hokje aankruist**. Wilt u als u het product nooit eet, het eerste hokje (*nooit of minder dan 1 keer per maand*) aankruisen. Als u een vergissing hebt gemaakt, kunt u het hokje zwart maken en een ander hokje aankruisen.

Voorbeeld 1.

Hoe vaak drinkt u melk?
Als uw antwoord 3 glazen melk per dag is, geeft u het als volgt aan:

**Zuivelproducten Per glas**	**nooit of minder dan 1 keer per maand**	**1 tot 3 keer per maand**	**1 keer per week**	**2 tot 4 keer per week**	**5 tot 6 keer per week**	**1 keer dag**	**2 tot 3 keer per dag**	**4 tot 5 keer per dag**	**meer dan 6 keer per dag**
Melk	❑	❑	❑	❑	❑	❑	☒	❑	❑

Open vragen

Wilt u bij vragen waar u iets opschrijft **in blokletters en binnen de omlijnde ruimte schrijven**?

Voorbeeld 2

Hoe vaak per week gebruikt u een warme maaltijd | 7 |

Hoeveelheden

Bij sommige onderdelen staat de hoeveelheid vermeld (bijvoorbeeld een glas of een schaaltje). Als er geen hoeveelheid wordt vermeld, wordt een gemiddelde portie bedoeld. Bij sommige onderdelen wordt ook gevraagd wat voor u een gemiddelde portie is. Hieronder volgt een voorbeeld van een vraag naar een gemiddelde portie.

Voorbeeld 3

Kruis aan welke foto weergeeft **hoeveel** u gemiddeld van een **samengesteld warm gerecht eet per maaltijd**.

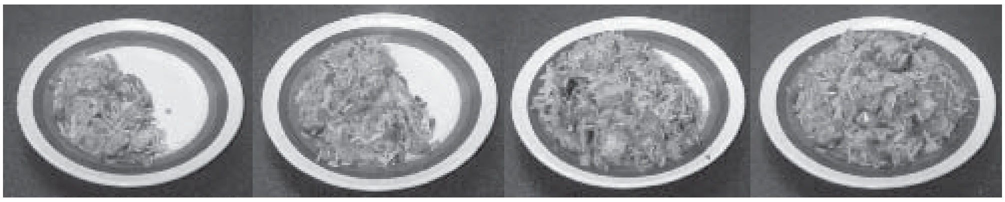

a. ❑ **b.** ❑ **c.** ❑ **d.** ☒

Einde instructie
Heel veel succes en alvast bedankt !!

A. WARME GERECHTEN

A1. **Hoe vaak** gebruikte u in de afgelopen 3 maanden de onderstaande **warme gerechten of maaltijden**?
(zelf gemaakt en kant en klaar)

Warme gerechten	nooit of minder dan 1 keer per maand	1 tot 3 keer per maand	1 keer per week	2 tot 4 keer per week	5 tot 6 keer per week	1 keer dag	2 tot 3 keer per dag	4 tot 5 keer per dag	meer dan 6 keer per dag
Aardappelen (gekookt, puree, stamppot)	❑	❑	❑	❑	❑	❑	❑	❑	❑
Gebakken aardappelen (geen patat)	❑	❑	❑	❑	❑	❑	❑	❑	❑
Patat	❑	❑	❑	❑	❑	❑	❑	❑	❑
Witte of gekleurde pasta (macaroni, spaghetti, lasangne)	❑	❑	❑	❑	❑	❑	❑	❑	❑
Volkoren pasta	❑	❑	❑	❑	❑	❑	❑	❑	❑
Pizza (1 hele)	❑	❑	❑	❑	❑	❑	❑	❑	❑
Graan producten (Tarly, Ebly, bulgur, couscous)	❑	❑	❑	❑	❑	❑	❑	❑	❑
Peulvruchten als basis (zoals chili con carne)	❑	❑	❑	❑	❑	❑	❑	❑	❑
Witte rijst (niet bij Chinees eten)	❑	❑	❑	❑	❑	❑	❑	❑	❑
Zilvervliesrijst	❑	❑	❑	❑	❑	❑	❑	❑	❑
Nasi, bami, Chinees eten	❑	❑	❑	❑	❑	❑	❑	❑	❑
Hartige taart (1 stuk)	❑	❑	❑	❑	❑	❑	❑	❑	❑
Kant en klaar maaltijden (diverse soorten)	❑	❑	❑	❑	❑	❑	❑	❑	❑
Soep als maaltijd (o.a. erwten-, bonensoep)	❑	❑	❑	❑	❑	❑	❑	❑	❑
Linzensoep als maaltijd	❑	❑	❑	❑	❑	❑	❑	❑	❑
Pannekoeken, flensjes	❑	❑	❑	❑	❑	❑	❑	❑	❑

Wilt u niet genoemde producten die u wel gebruikt, vermelden op bladzijde 26?

A2. Hoeveel **<u>dagen per week</u>** eet u gewoonlijk een of meerdere **warme maaltijden**?(zelf gemaakt of kant en klaar)

- ❑ Nooit, ga naar **A5**
- ❑ Minder dan 1 dag per week
- ❑ 1 tot 2 dagen dag per week
- ❑ 3 tot 4 dagen per week
- ❑ 5 tot 6 dagen per week
- ❑ 7 dagen per week

A3. Hoe vaak **<u>per dag</u>** eet u gewoonlijk een **warme maaltijd**? (zelf gemaakt of kant en klaar)

- ❑ Minder dan 1 keer per dag
- ❑ 1 keer per dag
- ❑ 1 tot 2 keer per dag
- ❑ 2 keer per dag
- ❑ 2 tot 3 keer per dag
- ❑ 3 keer per dag
- ❑ Meer dan 3 keer per dag

A4. Hoeveel **<u>dagen per week</u>** eet u een **kant en klare maaltijd**
`
(diepvries of vers) als warme maaltijd?
(van supermarkt of afhaalrestaurant)

- ❑ Nooit
- ❑ Minder dan 1 dag per week
- ❑ 1 dag per week
- ❑ 1 tot 2 dagen per week
- ❑ 3 tot 4 dagen per week
- ❑ 5 tot 7 dagen per week

A5. **Hoe vaak** gebruikte u in de afgelopen 3 maanden de onderstaande **soepen** tussendoor, vooraf aan een warme maaltijd of bij een brood maaltijd?

**Soepen (per kom)**	**nooit of minder dan 1 keer per maand**	**1 tot 3 keer per maand**	**1 keer per week**	**2 tot 4 keer per week**	**5 tot 6 keer per week**	**1 keer dag**	**2 tot 3 keer per dag**	**4 tot 5 keer per dag**	**meer dan 6 keer per dag**
Heldere soep zonder vlees of vulling (bouillon, cup a soep, etc.) (1 kom)	❑	❑	❑	❑	❑	❑	❑	❑	❑
Heldere soep met vlees (1 kom)	❑	❑	❑	❑	❑	❑	❑	❑	❑
Soep met vermicelli, rijst, macaroni of bloem zonder vlees (1 kom)	❑	❑	❑	❑	❑	❑	❑	❑	❑
Soep met vermicelli, rijst, macaroni of bloem met vlees (1 kom)	❑	❑	❑	❑	❑	❑	❑	❑	❑

A6. **Hoeveel aardappelen** eet u bij een gemiddelde warme maaltijd?

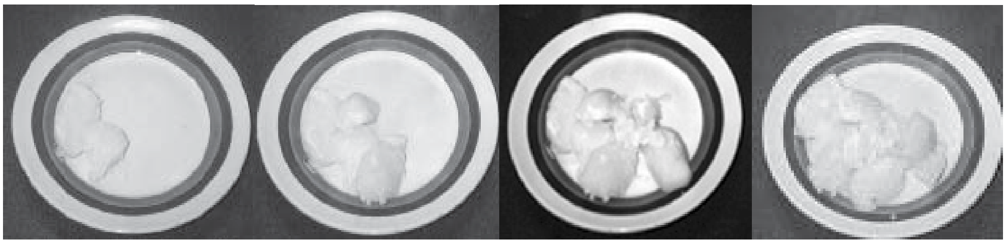

a. ❑ **b.** ❑ **c.** ❑ **d.** ❑

❑ Ik eet geen aardappelen

A7. **Hoeveel** eet u gemiddeld van een **eenpansgerecht of stoofschotel**?

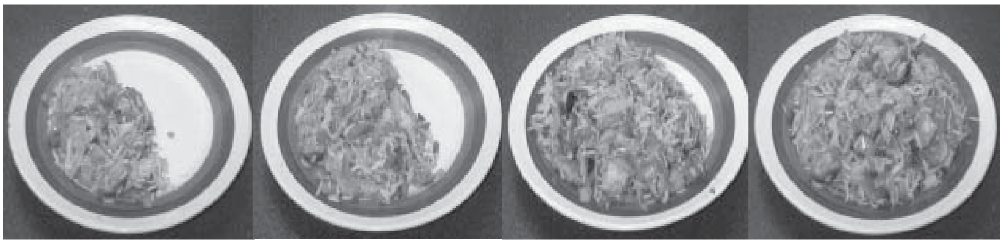

a. ❑ **b.** ❑ **c.** ❑ **d.** ❑

A8. **Hoeveel rijst** eet u bij een gemiddelde warme maaltijd?

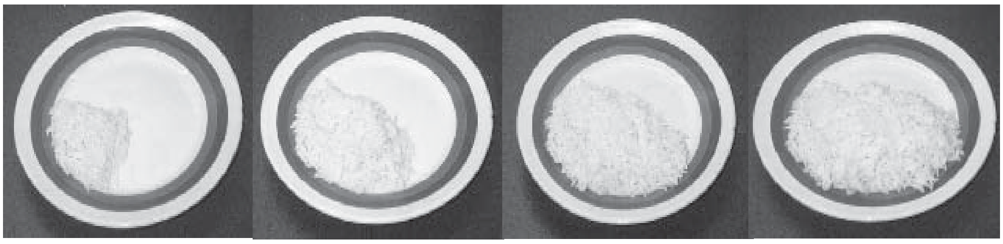

a. ❑ **b.** ❑ **c.** ❑ **d.** ❑

❑ Ik eet geen rijst

A9. Gebruikt u **mayonaise, fritessaus, slasaus of yogonaise**? (bij alle gerechten, meerdere antwoorden mogelijk)

- ❑ Ik gebruik geen van allen
- ❑ Mayonaise
- ❑ Fritessaus of slasaus
- ❑ Yogonaise

Wat is de **merknaam van de mayonaise, fritessaus, slasaus of yogonaise** die u gewoonlijk gebruikt?

A10. Wordt er **water** toegevoegd bij de bereiding van **jus**? (één antwoord mogelijk)

- ❑ Ik gebruik geen jus
- ❑ Nee
- ❑ Ja, alleen om af te blussen
- ❑ Ja, veel water
- ❑ Ja, de jus wordt ontvet

A11. **Hoe vaak** gebruikte u in de afgelopen 3 maanden de onderstaande **sauzen en bijgerechten** bij een warme maaltijd?

**Sauzen en bijgerechten**	**nooit of minder dan 1 keer per maand**	**1 tot 3 keer per maand**	**1 keer per week**	**2 tot 4 keer per week**	**5 tot 6 keer per week**	**1 keer dag**	**2 tot 3 keer per dag**	**4 tot 5 keer per dag**	**meer dan 6 keer per dag**
Mayonaise, fritesaus, etc. (1 eetlepel)	❑	❑	❑	❑	❑	❑	❑	❑	❑
Jus (1 eetlepel)	❑	❑	❑	❑	❑	❑	❑	❑	❑
Ketjap (1 eetlepel)	❑	❑	❑	❑	❑	❑	❑	❑	❑
Pindasaus (1 eetlepel)	❑	❑	❑	❑	❑	❑	❑	❑	❑
Groentesaus (groentebinder, room-, kaassaus) (1 eetlepel)	❑	❑	❑	❑	❑	❑	❑	❑	❑
Rode saus (chilis-, barbequesaus) (1 eetlepel)	❑	❑	❑	❑	❑	❑	❑	❑	❑
Knoflook-, bieslook-, whiskey- of mosterdsaus (1 eetlepel)	❑	❑	❑	❑	❑	❑	❑	❑	❑
Tomatensaus (gepelde tomaten, uit blik, puree en ketchup) (1 portie)	❑	❑	❑	❑	❑	❑	❑	❑	❑
Appelmoes (1 portie)	❑	❑	❑	❑	❑	❑	❑	❑	❑
Rabarber (1 portie)	❑	❑	❑	❑	❑	❑	❑	❑	❑
Atjar tjampoer (1 eetlepel)	❑	❑	❑	❑	❑	❑	❑	❑	❑
Sambal (1 eetlepel)	❑	❑	❑	❑	❑	❑	❑	❑	❑

B. GROENTEN

B1. Hoeveel **dagen per week** eet u **gekookte, gestoomde of roergebakken groenten**? (geen rauwe groenten zoals sla)

- ❑ Nooit, ga naar **B3**
- ❑ Minder dan 1 dag per week
- ❑ 1 tot 2 dagen per week
- ❑ 3 tot 4 dagen per week
- ❑ 5 tot 6 dagen per week
- ❑ 7 dagen per week

B2. Hoe vaak **per dag** eet u gewoonlijk **gekookte, gestoomde of roergebakken groente**?

- ❑ Minder dan 1 keer per dag
- ❑ 1 keer per dag
- ❑ 1 tot 2 keer per dag
- ❑ 2 keer per dag
- ❑ 2 tot 3 keer per dag
- ❑ 3 keer per dag
- ❑ Meer dan 3 keer per dag

B3. Hoeveel **dagen per week** eet u **sla of andere rauwe groenten**?

- ❑ Nooit, ga naar **B5**
- ❑ Minder dan 1 dag per week
- ❑ 1 tot 2 dagen per week
- ❑ 3 tot 4 dagen per week
- ❑ 5 tot 6 dagen per week
- ❑ 7 dagen per week

B4. Hoe vaak **per dag** eet u gewoonlijk **sla of andere rauwe groenten**?

- ❑ Minder dan 1 keer per dag
- ❑ 1 keer per dag
- ❑ 1 tot 2 keer per dag
- ❑ 2 keer per dag
- ❑ 2 tot 3 keer per dag
- ❑ 3 keer per dag
- ❑ Meer dan 3 keer per dag

B5. **Hoeveel groenten** eet u gemiddeld bij een warme maaltijd? (bereid of rauw)

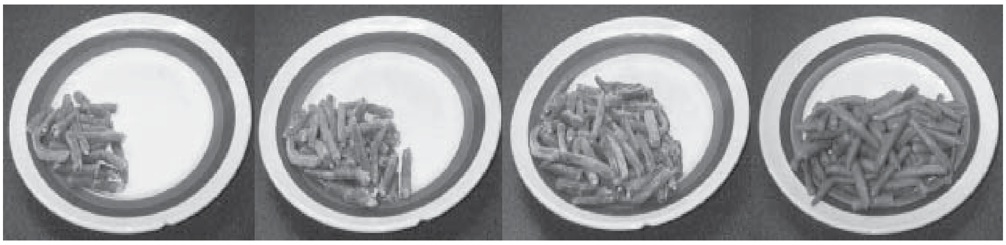

a. ❑ **b.** ❑ **c.** ❑ **d.** ❑

B6. **Hoe vaak** gebruikte u in de afgelopen 3 maanden de onderstaande **gekookte, gestoomde of roergebakken groenten**?

**Groenten (bereid)**	**nooit of minder dan 1 keer per maand**	**1 tot 3 keer per maand**	**1 keer per week**	**2 tot 4 keer per week**	**5 tot 6 keer per week**	**1 keer dag**	**2 tot 3 keer per dag**	**4 tot 5 keer per dag**	**meer dan 6 keer per dag**
Bloemkool	❑	❑	❑	❑	❑	❑	❑	❑	❑
Broccoli	❑	❑	❑	❑	❑	❑	❑	❑	❑
Spruitjes, kool (witte, rode, spits, chinese)	❑	❑	❑	❑	❑	❑	❑	❑	❑
Rode biet	❑	❑	❑	❑	❑	❑	❑	❑	❑
Snijbiet	❑	❑	❑	❑	❑	❑	❑	❑	❑
Sperziebonen, snijbonen, slabonen, peultjes	❑	❑	❑	❑	❑	❑	❑	❑	❑
Doperwten, tuinbonen	❑	❑	❑	❑	❑	❑	❑	❑	❑
Linzen	❑	❑	❑	❑	❑	❑	❑	❑	❑
Mais	❑	❑	❑	❑	❑	❑	❑	❑	❑
Witlof	❑	❑	❑	❑	❑	❑	❑	❑	❑
Prei	❑	❑	❑	❑	❑	❑	❑	❑	❑
Andijvie, spinazie, postelein, raapsteeltjes	❑	❑	❑	❑	❑	❑	❑	❑	❑
Gemengde roerbak groenten	❑	❑	❑	❑	❑	❑	❑	❑	❑
Wortelen, hutspot, winterpeen	❑	❑	❑	❑	❑	❑	❑	❑	❑
Boerenkool	❑	❑	❑	❑	❑	❑	❑	❑	❑
Zuurkool	❑	❑	❑	❑	❑	❑	❑	❑	❑

Wilt u niet genoemde producten die u wel gebruikt, vermelden op bladzijde 26?

B7. **Hoe vaak** gebruikte u in de afgelopen 3 maanden de onderstaande **rauwe groenten?** (tussendoor of bij een maaltijd)

**Groenten (rauw)**	**nooit of minder dan 1 keer per maand**	**1 tot 3 keer per maand**	**1 keer per week**	**2 tot 4 keer per week**	**5 tot 6 keer per week**	**1 keer dag**	**2 tot 3 keer per dag**	**4 tot 5 keer per dag**	**meer dan 6 keer per dag**
Kool (witte, rode, spits, chinese) (1 portie)	❑	❑	❑	❑	❑	❑	❑	❑	❑
Witlof (1 portie)	❑	❑	❑	❑	❑	❑	❑	❑	❑
Winterpeen (1stuks)	❑	❑	❑	❑	❑	❑	❑	❑	❑
Wortel (1 portie)	❑	❑	❑	❑	❑	❑	❑	❑	❑
Andijvie, spinazie (1 portie)	❑	❑	❑	❑	❑	❑	❑	❑	❑
Sla (ook gemengde soorten) (1portie)	❑	❑	❑	❑	❑	❑	❑	❑	❑
Komkommer (1/2 stuks)	❑	❑	❑	❑	❑	❑	❑	❑	❑
Bleekselderij (1 portie)	❑	❑	❑	❑	❑	❑	❑	❑	❑

B8. **Hoeveel kocht** u in de afgelopen 3 maanden van de onderstaande **groenten voor uw huishouden of gezin**?

Uien	__ __	stuks **per week**
Tomaten	__ __	stuks **per week**
Courgette	__ __	stuks **per maand**
Champignons	__ __	bakjes **per maand**
Alfafa, tauge	__ __	bakjes **per maand**
Paprika	__ __	stuks **per maand**
Aubergine	__ __	stuks **per maand**
Knoflook	__ __	teentjes **per maand**

Voor hoeveel mensen wordt gewoonlijk bij u thuis gekookt ?

__ __	**volwassenen**
__ __	**kinderen**

B9. Waarmee maakt u **sla en rauwkost** gewoonlijk aan? (meerdere antwoorden mogelijk)

	**Merk**
❑ Slasaus, fritessaus	
❑ Mayonaise	
❑ Yogonaise	
❑ Dressing	
❑ Slafris	
❑ Yoghurt of kwark	

❑ Azijn of olie

- ❑ Arachideolie ❑ Sesamolie ❑ Mais(kiem)olie
- ❑ Notenolie ❑ Zonnebloemolie
- ❑ Slaolie ❑ Olijfolie

❑ Anders of niet aangemaakt	

C. VLEES, VIS EN VEGETARISCHE PRODUCTEN

C1. Hoeveel **dagen per week** eet u **vlees** (alle soorten) bij een **Warme maaltijd**? (ook in gemengde gerechten)

- ❑ Nooit, ga naar **C7**
- ❑ Minder dan 1 dag per week
- ❑ 1 tot 2 dagen per week
- ❑ 3 tot 4 dagen per week
- ❑ 5 tot 6 dagen per week
- ❑ 7 dagen per week

C2. Hoe vaak **per dag** eet u gewoonlijk **vlees** bij een **warme maaltijd**? (ook in gemengde gerechten)

- ❑ Minder dan 1 keer per dag
- ❑ 1 keer per dag
- ❑ 1 tot 2 keer per dag
- ❑ 2 keer per dag
- ❑ 2 tot 3 keer per dag
- ❑ 3 keer per dag
- ❑ Meer dan 3 keer per dag

C3. **Hoeveel vlees** eet u bij een gemiddelde warme maaltijd?

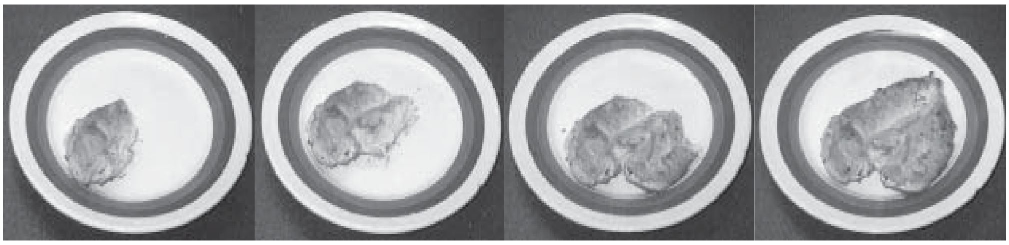

a. ❑ **b.** ❑ **c.** ❑ **d.** ❑

C4. Welke soort **vet of olie** gebruikt u voor het bakken van **vlees**?
(meerdere antwoorden mogelijk)

- ❑ Roomboter
- ❑ Margarine — ❑ Pakje ❑ Kuipje — **Merk**: [ ]
- ❑ Bak- en braad vet — ❑ Pakje ❑ Kuipje ❑ Fles — [ ]
- ❑ Olie, soort — ❑ Arachideolie ❑ Notenolie ❑ Slaolie ❑ Sesamolie ❑ Zonnebloemolie ❑ Olijfolie ❑ Mais(kiem)olie
- ❑ Anders [ ]

C5. Hoeveel **dagen per week** eet u **gegrild of geroosterd vlees** bij een warme maaltijd?

- ❑ Nooit
- ❑ Minder dan 1 dag per week
- ❑ 1 tot 2 dagen per week
- ❑ 3 tot 4 dagen per week
- ❑ 5 tot 6 dagen per week
- ❑ 7 dagen per week

C6. **Hoe vaak** gebruikte u in de afgelopen 3 maanden de onderstaande **vleessoorten** bij een warme maaltijd?

**Vlees**	nooit of minder dan 1 keer per maand	1 tot 3 keer per maand	1 keer per week	2 tot 4 keer per week	5 tot 6 keer per week	1 keer dag	2 tot 3 keer per dag	4 tot 5 keer per dag	meer dan 6 keer per dag
Kippengehakt	❑	❑	❑	❑	❑	❑	❑	❑	❑
Kippenlever	❑	❑	❑	❑	❑	❑	❑	❑	❑
Kip, kalkoen, andere gevogelte (geen gehakt of lever)	❑	❑	❑	❑	❑	❑	❑	❑	❑
Runder- of kalfslever	❑	❑	❑	❑	❑	❑	❑	❑	❑
Kalfsvlees	❑	❑	❑	❑	❑	❑	❑	❑	❑
Biefstuk of lap, rosbief, tartaar, lendenlap	❑	❑	❑	❑	❑	❑	❑	❑	❑
Runderlap, rundervink of rundergehakt (geen half-om-half gehakt)	❑	❑	❑	❑	❑	❑	❑	❑	❑
Rookworst	❑	❑	❑	❑	❑	❑	❑	❑	❑
Half-om-half gehakt	❑	❑	❑	❑	❑	❑	❑	❑	❑
Varkenslever	❑	❑	❑	❑	❑	❑	❑	❑	❑

**Vlees (vervolg)**	nooit of minder dan 1 keer per maand	1 tot 3 keer per maand	1 keer per week	2 tot 4 keer per week	5 tot 6 keer per week	1 keer dag	2 tot 3 keer per dag	4 tot 5 keer per dag	meer dan 6 keer per dag
Karbonade, varkenslappen of -rollade, hamlappen	❑	❑	❑	❑	❑	❑	❑	❑	❑
Speklap, rookspek, spekjes (ook in gerechten)	❑	❑	❑	❑	❑	❑	❑	❑	❑
Verse worst, saucijs, hamburger, slavink, varkensgehakt (ook in gerechten)	❑	❑	❑	❑	❑	❑	❑	❑	❑
Varkenshaas, fricandeau, schnitzel	❑	❑	❑	❑	❑	❑	❑	❑	❑
Schapenvlees	❑	❑	❑	❑	❑	❑	❑	❑	❑
Paardenvlees	❑	❑	❑	❑	❑	❑	❑	❑	❑
Lamsvlees	❑	❑	❑	❑	❑	❑	❑	❑	❑
Shoarmavlees, Thaise reepjes	❑	❑	❑	❑	❑	❑	❑	❑	❑
Frikandel, kroket, etc.	❑	❑	❑	❑	❑	❑	❑	❑	❑

C7. Hoeveel **<u>dagen per week</u>** eet u **vis** bij een **warme maaltijd**? (alle soorten)

- ❑ Nooit, ga naar **C13**
- ❑ Minder dan 1 dag per week
- ❑ 1 tot 2 dagen per week
- ❑ 3 tot 4 dagen per week
- ❑ 5 tot 6 dagen per week
- ❑ 7 dagen per week

C8. Hoe vaak **<u>per dag</u>** eet u **vis** bij een **warme maaltijd**? (alle soorten)

- ❑ Minder dan 1 keer per dag
- ❑ 1 keer per dag
- ❑ 1 tot 2 keer per dag
- ❑ 2 keer per dag
- ❑ 2 tot 3 keer per dag
- ❑ 3 keer per dag
- ❑ Meer dan 3 keer per dag

C9. **Hoe vaak** gebruikte u in de afgelopen 3 maanden de onderstaande **vissoorten**? (bij alle maaltijden)

**Vis**	**nooit of minder dan 1 keer per maand**	**1 tot 3 keer per maand**	**1 keer per week**	**2 tot 4 keer per week**	**5 tot 6 keer per week**	**1 keer dag**	**2 tot 3 keer per dag**	**4 tot 5 keer per dag**	**meer dan 6 keer per dag**
Vissticks, visburger, visschnitzel, lekkerbekje, kibbeling	❑	❑	❑	❑	❑	❑	❑	❑	❑
Zalm, haring, makreel, paling, sardines, heilbot, bokking	❑	❑	❑	❑	❑	❑	❑	❑	❑
Kabeljauw, schol, zeewolf, tong, tonijn, wijting, schelvis	❑	❑	❑	❑	❑	❑	❑	❑	❑
Forel, ansjovis, poon	❑	❑	❑	❑	❑	❑	❑	❑	❑
Schelvislever	❑	❑	❑	❑	❑	❑	❑	❑	❑
Kuit, hom	❑	❑	❑	❑	❑	❑	❑	❑	❑
Krab, garnalen, mosselen, kreeft	❑	❑	❑	❑	❑	❑	❑	❑	❑

C10. **Hoeveel vis** eet u bij een gemiddelde maaltijd?

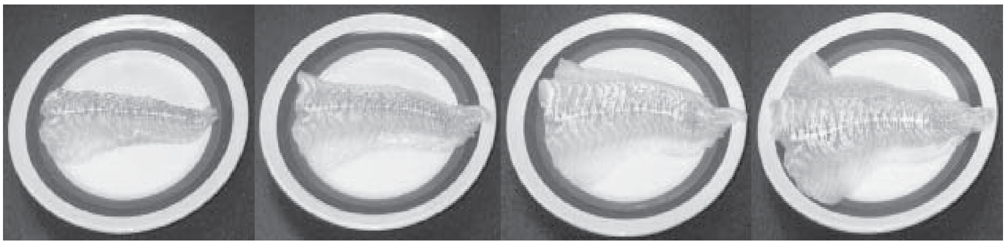

a. ❑ **b.** ❑ **c.** ❑ **d.** ❑

C11. Hoe **bereid** u gewoonlijk **vis**?
(meerdere antwoorden mogelijk)

- ❑ Grillen
- ❑ Stomen, koken
- ❑ Bakken
- ❑ Magnetron

C12. Welk soort **vet of olie** gebruikt u voor het **bereiden van vis**?
(meerdere antwoorden mogelijk)

- ❑ Roomboter
- ❑ Margarine — ❑ Pakje ❑ Kuipje — **Merk**: ______
- ❑ Bak- en braad vet — ❑ Pakje ❑ Kuipje ❑ Fles — ______
- ❑ Olie, soort — ❑ Arachideolie ❑ Notenolie ❑ Slaolie ❑ Sesamolie ❑ Zonnebloemolie ❑ Olijfolie ❑ Mais(kiem)olie
- ❑ Anders ______

C13. Hoeveel **<u>dagen per week</u>** eet u een **vegetarische maaltijd** als **warme maaltijd**? (Hiermee wordt een hoofdmaaltijd bedoeld zonder vlees of vis)

- ❑ Nooit, ga naar **D1**
- ❑ Minder dan 1 dag per week
- ❑ 1 tot 2 dagen per week
- ❑ 3 tot 4 dagen per week
- ❑ 5 tot 6 dagen per week
- ❑ 7 dagen per week

C14. Hoe vaak **<u>per dag</u>** eet u een **vegetarische maaltijd** als **warme maaltijd**?

- ❑ Minder dan 1 keer per dag
- ❑ 1 keer per dag
- ❑ 1 tot 2 keer per dag
- ❑ 2 keer per dag
- ❑ 2 tot 3 keer per dag
- ❑ 3 keer per dag
- ❑ Meer dan 3 keer per dag

C15. **Hoe vaak** gebruikte u in de afgelopen 3 maanden de volgende **vegetarische producten**?

**Vegetarische producten**	**nooit of minder dan 1 keer per maand**	**1 tot 3 keer per maand**	**1 keer per week**	**2 tot 4 keer per week**	**5 tot 6 keer per week**	**1 keer dag**	**2 tot 3 keer per dag**	**4 tot 5 keer per dag**	**meer dan 6 keer per dag**
Quom	❑	❑	❑	❑	❑	❑	❑	❑	❑
Seitan	❑	❑	❑	❑	❑	❑	❑	❑	❑
Sojabrokjes	❑	❑	❑	❑	❑	❑	❑	❑	❑
Tofu, tahoe	❑	❑	❑	❑	❑	❑	❑	❑	❑
Tempeh	❑	❑	❑	❑	❑	❑	❑	❑	❑
Vegetarische of groenteburgers	❑	❑	❑	❑	❑	❑	❑	❑	❑
Kaas (in plaats van vlees)	❑	❑	❑	❑	❑	❑	❑	❑	❑
Noten (in plaats van vlees)	❑	❑	❑	❑	❑	❑	❑	❑	❑

C16. Welke **soort vet of olie** gebruikt u voor het bereiden van **vegetarische producten**? (meerdere antwoorden mogelijk)

- ❑ Roomboter

Merk

- ❑ Margarine — ❑ Pakje ❑ Kuipje — [ ]
- ❑ Bak- en braad vet — ❑ Pakje ❑ Kuipje ❑ Fles — [ ]
- ❑ Olie, soort — ❑ Arachideolie ❑ Notenolie ❑ Slaolie ❑ Sesamolie ❑ Zonnebloemolie ❑ Olijfolie ❑ Mais(kiem)olie
- ❑ Anders [ ]

D. BROOD EN BELEG

D1. **Hoe vaak** gebruikte u in de afgelopen 3 maanden de onderstaande **broodsoorten (sneetjes, bolletjes, stukjes)**? (totaal, bij alle maaltijden)

**Brood (per snee, bolletje of stuk)**	**nooit of minder dan 1 keer per maand**	**1 tot 3 keer per maand**	**1 keer per week**	**2 tot 4 keer per week**	**5 tot 6 keer per week**	**1 keer dag**	**2 tot 3 keer per dag**	**4 tot 5 keer per dag**	**meer dan 6 keer per dag**
Wit brood (1 snee)	❑	❑	❑	❑	❑	❑	❑	❑	❑
Tarwe- en fijn volkoren brood (1 snee)	❑	❑	❑	❑	❑	❑	❑	❑	❑
Grof volkoren brood, meergranen (1 snee)	❑	❑	❑	❑	❑	❑	❑	❑	❑
Krentenbrood, rozijnen brood, mueslibrood (1 snee)	❑	❑	❑	❑	❑	❑	❑	❑	❑
Krentenbol, mueslibol (1 bol)	❑	❑	❑	❑	❑	❑	❑	❑	❑
Croissant (1 stuks)	❑	❑	❑	❑	❑	❑	❑	❑	❑
Wit stokbrood (2 stukjes)	❑	❑	❑	❑	❑	❑	❑	❑	❑
Bruin en volkoren stokbrood (2 stukjes)	❑	❑	❑	❑	❑	❑	❑	❑	❑
Crackers, knäckebröt (1 stuks)	❑	❑	❑	❑	❑	❑	❑	❑	❑
Beschuit (1 stuks)	❑	❑	❑	❑	❑	❑	❑	❑	❑
Ontbijtkoek (1 plak)	❑	❑	❑	❑	❑	❑	❑	❑	❑
Roggebrood (1 plak)	❑	❑	❑	❑	❑	❑	❑	❑	❑

Wilt u niet genoemde producten die u wel gebruikt, vermelden op bladzijde 26?

D2. **Hoeveel sneetjes, bolletjes of stukjes** brood eet u **<u>in totaal per dag</u>**? (het gaat om het totaal bij alle maaltijden)

- ❑ Minder dan 1 per dag
- ❑ 1 per dag
- ❑ 2 tot 3 per dag
- ❑ 4 tot 6 per dag
- ❑ 7 tot 9 per dag
- ❑ 10 tot 12 per dag
- ❑ Meer dan 12 per dag

D3. Eet u gewoonlijk **brood met zaden**? (meerdere antwoorden mogelijk)

- ❑ Nee
- ❑ Ja, met sesamzaad
- ❑ Ja, met pompoenpitten
- ❑ Ja, met maanzaad
- ❑ Ja, met lijnzaad
- ❑ Ja, met zonnebloempitten
- ❑ Ja, met gemengde zaden

D4. **Hoe vaak** gebruikt u **boter of margarine** op brood, crackers, ontbijtkoek etc.? (één antwoord mogelijk)

- ❑ Nooit, ga naar **D6**
- ❑ Zelden
- ❑ Soms
- ❑ Meestal
- ❑ Altijd

D5. Welke **boter of margarine smeert** u gewoonlijk op brood? (één antwoord mogelijk)

- ❑ Margarine (kuipje)
- ❑ Margarine (pakje)
- ❑ Roomboter
- ❑ Halvarine
- ❑ Halfvolle roomboter
- ❑ Dieetmargarine
- ❑ Anders, namelijk:

Wat is de **merknaam van de boter, (dieet) margarine of halvarine** die u gewoonlijk gebruikt?

D6. Welke van de volgende **vleeswaren** gebruikt u **één keer per maand of vaker**? (meerdere antwoorden mogelijk)

- ❑ Ik eet geen vleeswaren, ga naar **D7**
- ❑ Gebraden gehakt
- ❑ Rosbief
- ❑ Ontbijtspek
- ❑ Kipfilet
- ❑ Tongeworst
- ❑ Rookvlees
- ❑ Biefstuk, tartaar
- ❑ Casselerrib
- ❑ Boterhamworst, gekookte worst, palingworst
- ❑ Corned beef
- ❑ Bloedworst
- ❑ Rauwe ham
- ❑ Fricandeau
- ❑ Pekelvlees
- ❑ Lever (worst), paté
- ❑ Zure zult
- ❑ Salami, cervelaat
- ❑ (Achter) Ham
- ❑ Anders, namelijk

D7. Hoe veel **boterhammen of brood vervangers** gebruikte u in de afgelopen 3 maanden met de volgende **beleg** soorten?

**Aantal boterhammen of brood vervangers met: (per snee, bolletje of stukje)**	**nooit of minder dan 1 keer per maand**	**1 tot 3 keer per maand**	**1 keer per week**	**2 tot 4 keer per week**	**5 tot 6 keer per week**	**1 keer dag**	**2 tot 3 keer per dag**	**4 tot 5 keer per dag**	**meer dan 6 keer per dag**
Suikervrije jam	❑	❑	❑	❑	❑	❑	❑	❑	❑
Honing, suiker, jam, vruchtenhagel	❑	❑	❑	❑	❑	❑	❑	❑	❑
Appelstroop	❑	❑	❑	❑	❑	❑	❑	❑	❑
Hazelnootpasta	❑	❑	❑	❑	❑	❑	❑	❑	❑
Hagelslag, vlokken, kokosbrood, chocoladepasta	❑	❑	❑	❑	❑	❑	❑	❑	❑
Pindakaas, notenpasta	❑	❑	❑	❑	❑	❑	❑	❑	❑
Tahin	❑	❑	❑	❑	❑	❑	❑	❑	❑
Marmite	❑	❑	❑	❑	❑	❑	❑	❑	❑
Magere kaas, magere smeerkaas (20-30+) huttenkäse, cottage cheese	❑	❑	❑	❑	❑	❑	❑	❑	❑
Gewone kaas, gewone smeerkaas (40-48+)	❑	❑	❑	❑	❑	❑	❑	❑	❑
Zachte buitenlandse kaas (brie, Franse, room, of kruidenkaas) (40-60+)	❑	❑	❑	❑	❑	❑	❑	❑	❑
Geitenkaas, feta	❑	❑	❑	❑	❑	❑	❑	❑	❑
Sandwich spread	❑	❑	❑	❑	❑	❑	❑	❑	❑
Natriumarme kaas	❑	❑	❑	❑	❑	❑	❑	❑	❑
Natriumarme vleeswaren	❑	❑	❑	❑	❑	❑	❑	❑	❑
Salade (huzaren, zalm, eier, kip-kerrie, etc.)	❑	❑	❑	❑	❑	❑	❑	❑	❑
Rauwkost	❑	❑	❑	❑	❑	❑	❑	❑	❑
Vleeswaren (alle soorten)	❑	❑	❑	❑	❑	❑	❑	❑	❑

E. ZUIVELPRODUCTEN

E1. Hoe vaak gebruikte u in de afgelopen 3 maanden de onderstaande **zuivel producten**?
(om te drinken, te eten of bij de bereiding van maaltijden)

**Zuivelproducten**	**nooit of minder dan 1 keer per maand**	**1 tot 3 keer per maand**	**1 keer per week**	**2 tot 4 keer per week**	**5 tot 6 keer per week**	**1 keer dag**	**2 tot 3 keer per dag**	**4 tot 5 keer per dag**	**meer dan 6 keer per dag**
Volle, halfvolle of magere melk (1 glas)	❑	❑	❑	❑	❑	❑	❑	❑	❑
Karnemelk (1 glas)	❑	❑	❑	❑	❑	❑	❑	❑	❑
Sojamelk (1 glas)	❑	❑	❑	❑	❑	❑	❑	❑	❑
Chocolademelk (warm of koud) (1 glas)	❑	❑	❑	❑	❑	❑	❑	❑	❑
Cacao (warm of koud) (1 glas)	❑	❑	❑	❑	❑	❑	❑	❑	❑
Melk met smaak (1 glas) (aardbei, banaan, etc.)	❑	❑	❑	❑	❑	❑	❑	❑	❑
Ontbijtdranken met melk of yoghurt, zoals Goedenmorgen	❑	❑	❑	❑	❑	❑	❑	❑	❑
Drink yoghurt (yoki, Vifit, etc.) (1 glas)	❑	❑	❑	❑	❑	❑	❑	❑	❑
Yoghurt, Biogarde (1 schaaltje)	❑	❑	❑	❑	❑	❑	❑	❑	❑
Kwark (1 schaaltje)	❑	❑	❑	❑	❑	❑	❑	❑	❑
Sojatoetje (Alpro) (1 schaaltje)	❑	❑	❑	❑	❑	❑	❑	❑	❑
Vla (1 schaaltje)	❑	❑	❑	❑	❑	❑	❑	❑	❑
Pudding (1 schaaltje)	❑	❑	❑	❑	❑	❑	❑	❑	❑
(Slag)roomijs, milkshake (1 portie) (geen waterijs)	❑	❑	❑	❑	❑	❑	❑	❑	❑
Slagroom, zure room, crème fraiche (1 eetlepel)	❑	❑	❑	❑	❑	❑	❑	❑	❑

E2. Welke **soorten melk en yoghurt** gebruikt u gewoonlijk?
(meerdere antwoorden mogelijk)

❑ Ik gebruik geen melk of yoghurt
❑ Magere producten met vruchten (kant en klaar)
❑ Magere producten naturel
❑ Halfvolle producten met vruchten (kant en klaar)
❑ Halfvolle producten naturel
❑ Volle producten met vruchten (kant en klaar)
❑ Volle producten naturel
❑ Producten met extra calcium
❑ Producten met zoetstoffen
❑ Producten met vitaminen

E3. Gebruikt u **suiker, honing of zoetstof** in uw **yoghurt of kwark**?
(1 eetlepel komt overeen met 3 theelepels)

- ❑ Niet van toepassing
- ❑ Nee
- ❑ Ja, suiker of honing ☐☐ theelepels **per schaaltje**
- ❑ Ja, zoetstof ☐☐ theelepels **per schaaltje**

E4. **Hoeveel eieren** eet u gemiddeld per week?

- ❑ 0 per week, ga naar **E6**
- ❑ Minder dan 1 per week
- ❑ 1 per week
- ❑ 1 tot 2 per week
- ❑ 3 tot 5 per week
- ❑ 6 tot 8 per week
- ❑ 9 tot 11 per week
- ❑ 12 tot 14 per week
- ❑ Meer dan 14 per week

E5. Hoe **bereid** u gewoonlijk de **eieren**?

- ❑ Bakken
- ❑ Koken
- ❑ Zowel koken als bakken

E6. **Hoe vaak** gebruikte u in de afgelopen 3 maanden de volgende **producten**?
(ook als toevoeging aan melk, yoghurt, salades of maaltijden)

**Toevoegingen**	nooit of minder dan 1 keer per maand	1 tot 3 keer per maand	1 keer per week	2 tot 4 keer per week	5 tot 6 keer per week	1 keer dag	2 tot 3 keer per dag	4 tot 5 keer per dag	meer dan 6 keer per dag
Muesli, cruesli (40 gram)	❑	❑	❑	❑	❑	❑	❑	❑	❑
Cornflakes, Kellog's ontbijtgranen (40 gram)	❑	❑	❑	❑	❑	❑	❑	❑	❑
Havermout (1 portie)	❑	❑	❑	❑	❑	❑	❑	❑	❑
Brinta, Bambix (1 portie)	❑	❑	❑	❑	❑	❑	❑	❑	❑
Zemelen (1 eetlepel)	❑	❑	❑	❑	❑	❑	❑	❑	❑
Tarwekiemen (1 eetlepel)	❑	❑	❑	❑	❑	❑	❑	❑	❑
Zonnebloempitten (1 eetlepel)	❑	❑	❑	❑	❑	❑	❑	❑	❑
Pijnboompitten (1 eetlepel)	❑	❑	❑	❑	❑	❑	❑	❑	❑
Lijnzaad (1 eetlepel)	❑	❑	❑	❑	❑	❑	❑	❑	❑
Diksap, ongezoet ,geconcentreerd vruchtensap (1 eetlepel)	❑	❑	❑	❑	❑	❑	❑	❑	❑
Rozenbottelsiroop (Roosvice, Karvan Cevitam) (1 eetlepel)	❑	❑	❑	❑	❑	❑	❑	❑	❑

F. FRUIT

F1. **Hoeveel stuks of porties fruit** eet u gemiddeld **per dag**?
(het gaat alleen over het eten van fruit in de afgelopen 3 maanden)

- ❑ Ik gebruik geen fruit
- ❑ Minder dan 1 ***per week***
- ❑ 1 tot 2 per week
- ❑ 3 tot 4 per week
- ❑ 5 tot 6 per week
- ❑ 1 tot 2 ***per dag***
- ❑ 3 tot 4 per dag
- ❑ 5 of meer per dag

F2. **Hoe vaak** gebruikte in de afgelopen 3 maanden de volgende **fruitsoorten**?
(ook geperst, op brood en in toetjes)

**Fruit**	**nooit of minder dan 1 keer per maand**	**1 tot 3 keer per maand**	**1 keer per week**	**2 tot 4 keer per week**	**5 tot 6 keer per week**	**1 keer dag**	**2 tot 3 keer per dag**	**4 tot 5 keer per dag**	**meer dan 6 keer per dag**
Mandarijn (1 stuks)	❑	❑	❑	❑	❑	❑	❑	❑	❑
Sinaasappel, grapefruit (1 stuks)	❑	❑	❑	❑	❑	❑	❑	❑	❑
Citroen, limoen (1 stuks)	❑	❑	❑	❑	❑	❑	❑	❑	❑
Banaan (1 stuks)	❑	❑	❑	❑	❑	❑	❑	❑	❑
Kiwi (1 stuks)	❑	❑	❑	❑	❑	❑	❑	❑	❑
Appel (1 stuks)	❑	❑	❑	❑	❑	❑	❑	❑	❑
Peer (1 stuks)	❑	❑	❑	❑	❑	❑	❑	❑	❑
Mango (1/2 stuks)	❑	❑	❑	❑	❑	❑	❑	❑	❑
Avocado (1/2 stuks)	❑	❑	❑	❑	❑	❑	❑	❑	❑
Perziken, nectarines (1 stuks)	❑	❑	❑	❑	❑	❑	❑	❑	❑
Abrikozen (1 stuks)	❑	❑	❑	❑	❑	❑	❑	❑	❑
Pruimen (1 stuks)	❑	❑	❑	❑	❑	❑	❑	❑	❑
Aardbeien, frambozen, bramen, bessen (1 schaaltje)	❑	❑	❑	❑	❑	❑	❑	❑	❑
Druiven, kersen (1 schaaltje)	❑	❑	❑	❑	❑	❑	❑	❑	❑
Ananas, meloen (1 kwart), watermeloen (1 portie)	❑	❑	❑	❑	❑	❑	❑	❑	❑
Fruit uit blik (1 portie)	❑	❑	❑	❑	❑	❑	❑	❑	❑

G. DRANKEN

G1. **Hoe vaak** dronk u in de afgelopen 3 maanden een kopje of glas van de volgende **dranken**?
(NB 1 blikje is 2 glazen)

**Dranken niet-alcoholistisch (kopje of glas)**	**nooit of minder dan 1 keer per maand**	**1 tot 3 keer per maand**	**1 keer per week**	**2 tot 4 keer per week**	**5 tot 6 keer per week**	**1 keer dag**	**2 tot 3 keer per dag**	**4 tot 5 keer per dag**	**meer dan 6 keer per dag**
Espresso	❑	❑	❑	❑	❑	❑	❑	❑	❑
Cappucino	❑	❑	❑	❑	❑	❑	❑	❑	❑
Koffie met caffeïne	❑	❑	❑	❑	❑	❑	❑	❑	❑
Koffie zonder caffeïne (o.a. Décafe)	❑	❑	❑	❑	❑	❑	❑	❑	❑
Gewone thee (ook met smaak)	❑	❑	❑	❑	❑	❑	❑	❑	❑
Groene thee	❑	❑	❑	❑	❑	❑	❑	❑	❑
Kruidenthee	❑	❑	❑	❑	❑	❑	❑	❑	❑
Sinaasappelsap, grapefruitsap uit pak	❑	❑	❑	❑	❑	❑	❑	❑	❑
Overige vruchtensappen (appelsap, druivensap, etc.) uit pak	❑	❑	❑	❑	❑	❑	❑	❑	❑
Vruchtensappen zelf bereid	❑	❑	❑	❑	❑	❑	❑	❑	❑
Tomatensap, groentesap	❑	❑	❑	❑	❑	❑	❑	❑	❑
Ontbijtdranken zonder melk of yoghurt, zoals HERO	❑	❑	❑	❑	❑	❑	❑	❑	❑
Sportdranken (AA, Extran)	❑	❑	❑	❑	❑	❑	❑	❑	❑
Frisdranken (geen light) (Cola, Fanta, Sprite, etc.)	❑	❑	❑	❑	❑	❑	❑	❑	❑
Light frisdranken (Cola light, Fanta light, Sprite light)	❑	❑	❑	❑	❑	❑	❑	❑	❑
Limonadesiroop	❑	❑	❑	❑	❑	❑	❑	❑	❑
Mineraalwater, bronwater	❑	❑	❑	❑	❑	❑	❑	❑	❑
Leidingwater	❑	❑	❑	❑	❑	❑	❑	❑	❑

G2. Gebruikt u **suiker, honing of zoetjes in uw koffie**? (1 eetlepel komt overeen met 3 theelepels)

- ❑ Ik drink geen koffie
- ❑ Nee
- ❑ Ja, suiker of honing [|] theelepels **per kopje**
- ❑ Ja, zoetstof [|] zoetjes **per kopje**

G3. Gebruikt u **melk in uw koffie**? (één antwoord mogelijk)

- ❑ Ik drink geen koffie
- ❑ Nee
- ❑ Ja, halfvolle melk
- ❑ Ja, volle koffiemelk
- ❑ Ja, dagverse melk scheutje
- ❑ Ja, dagverse melk driekwart
- ❑ Ja, melkpoeder
- ❑ Ja, halfvolle koffiemelk
- ❑ Ja, room
- ❑ Ja, slagroom

G4. Gebruikt u **suiker, honing of zoetjes in uw thee**? (1 eetlepel komt overeen met 3 theelepels)

- ❑ Ik drink geen thee
- ❑ Nee
- ❑ Ja, suiker of honing [|] theelepels **per kopje**
- ❑ Ja, zoetstof [|] zoetjes **per kopje**

G5. Gebruikt u **melk in uw thee**? (één antwoord mogelijk)

- ❑ Ik drink geen thee
- ❑ Nee
- ❑ Ja

G6. Gebruikt u wel eens **alcoholische dranken**? (alle soorten)

- ❑ Nee nooit, ga naar **H1**
- ❑ Ja, gemiddeld minder dan 1 glas per week
- ❑ Ja, gemiddeld 1 tot 3 glazen per week
- ❑ Ja, gemiddeld 4 tot 6 glazen per week
- ❑ Ja, gemiddeld 1 glas per dag
- ❑ Ja, gemiddeld 1 tot 3 glazen per dag
- ❑ Ja, gemiddeld meer dan 3 glazen per dag

G7. **Hoe vaak** dronk u in de afgelopen 3 maanden een glas van de onderstaande **alcoholische dranken**?

**Dranken alcoholistisch (1 glas)**	**nooit of minder dan 1 keer per maand**	**1 tot 3 keer per maand**	**1 keer per week**	**2 tot 4 keer per week**	**5 tot 6 keer per week**	**1 keer dag**	**2 tot 3 keer per dag**	**4 tot 5 keer per dag**	**meer dan 6 keer per dag**
Sherry, port, martini, vermouth	❑	❑	❑	❑	❑	❑	❑	❑	❑
Rode wijn	❑	❑	❑	❑	❑	❑	❑	❑	❑
Witte wijn	❑	❑	❑	❑	❑	❑	❑	❑	❑
Bier normaal	❑	❑	❑	❑	❑	❑	❑	❑	❑
Bier alcoholarm, -vrij, malt	❑	❑	❑	❑	❑	❑	❑	❑	❑
Likeur (Bessen jenever, Campari, Pasoa,	❑	❑	❑	❑	❑	❑	❑	❑	❑
Pisang Ambon, Malibu, Baileys, etc.)	❑	❑	❑	❑	❑	❑	❑	❑	❑
Berenburg, Jäger-meister, cognac, brandewijn	❑	❑	❑	❑	❑	❑	❑	❑	❑
Jenever, vieux, whis-key, vodka	❑	❑	❑	❑	❑	❑	❑	❑	❑
Mixdrankjes (Bacardi Breezer, etc.)	❑	❑	❑	❑	❑	❑	❑	❑	❑

Wilt u niet genoemde producten die u wel gebruikt, vermelden op bladzijde 26?

H. TUSSENDOORTJES

H1. **Hoe vaak** gebruikte u in de afgelopen 3 maanden de onderstaande **zoete tussendoortjes**?

**Zoete tussendoortjes**	**nooit of minder dan 1 keer per maand**	**1 tot 3 keer per maand**	**1 keer per week**	**2 tot 4 keer per week**	**5 tot 6 keer per week**	**1 keer dag**	**2 tot 3 keer per dag**	**4 tot 5 keer per dag**	**meer dan 6 keer per dag**
Chocolade (1 bonbon, 2 stukjes)	❑	❑	❑	❑	❑	❑	❑	❑	❑
Chocoladereep (1 stuks)	❑	❑	❑	❑	❑	❑	❑	❑	❑
Candy bar (1 reep) (Nuts, Mars, Bounty, etc.)	❑	❑	❑	❑	❑	❑	❑	❑	❑
Vruchtentaart (1 punt)	❑	❑	❑	❑	❑	❑	❑	❑	❑
Overige taart en gebak (slagroom, mokka) (1 punt)	❑	❑	❑	❑	❑	❑	❑	❑	❑
Cake, grote koeken, (eierkoek,	❑	❑	❑	❑	❑	❑	❑	❑	❑
stroopwafels, gevulde koek) (1 plak of stuks)	❑	❑	❑	❑	❑	❑	❑	❑	❑
Biscuit, kaakje (1 stuks)	❑	❑	❑	❑	❑	❑	❑	❑	❑
Koekje (1 stuks)	❑	❑	❑	❑	❑	❑	❑	❑	❑
Mueslikoeken, volkoren koeken (liga, sultana, energierepen) (1 stuks)	❑	❑	❑	❑	❑	❑	❑	❑	❑
Suikervrije drop (2 stuks tot 1 handje)	❑	❑	❑	❑	❑	❑	❑	❑	❑
Drop (2 stuks tot 1 handje)	❑	❑	❑	❑	❑	❑	❑	❑	❑
Suikervrije snoep (2 stuks tot 1 handje)	❑	❑	❑	❑	❑	❑	❑	❑	❑
Snoep (pepermunt, zuurtjes, etc.) (2 stuks tot 1 handje)	❑	❑	❑	❑	❑	❑	❑	❑	❑
Rozijnen, krenten, dadels, vijgen, tutti frutti, gedroogde abrikozen (1 handje)	❑	❑	❑	❑	❑	❑	❑	❑	❑
Gedroogde pruimen (1 handje)	❑	❑	❑	❑	❑	❑	❑	❑	❑
Waterijs (1 stuks)	❑	❑	❑	❑	❑	❑	❑	❑	❑

H2. **Hoe vaak** gebruikte u in de afgelopen 3 maanden de onderstaande **hartige tussendoortjes**?

**Hartige tussendoortjes**	**nooit of minder dan 1 keer per maand**	**1 tot 3 keer per maand**	**1 keer per week**	**2 tot 4 keer per week**	**5 tot 6 keer per week**	**1 keer dag**	**2 tot 3 keer per dag**	**4 tot 5 keer per dag**	**meer dan 6 keer per dag**
Pinda's studentenhaver, borrelnootjes (1 handje)	❑	❑	❑	❑	❑	❑	❑	❑	❑
Japanse mix, krokante rijstzoutjes (1 handje)	❑	❑	❑	❑	❑	❑	❑	❑	❑
Overige noten (1 handje)	❑	❑	❑	❑	❑	❑	❑	❑	❑
Chips, zoutjes (1 handje)	❑	❑	❑	❑	❑	❑	❑	❑	❑
Frikandel, kroket, etc. (1 stuks)	❑	❑	❑	❑	❑	❑	❑	❑	❑
Knacksworst (1 stuks)	❑	❑	❑	❑	❑	❑	❑	❑	❑
Sate, bitterballen, gehaktbal, hamburger (1 portie)	❑	❑	❑	❑	❑	❑	❑	❑	❑
Patat (als tussendoor)	❑	❑	❑	❑	❑	❑	❑	❑	❑
Kaas (1 blokje)	❑	❑	❑	❑	❑	❑	❑	❑	❑
Toast (1 stuks)	❑	❑	❑	❑	❑	❑	❑	❑	❑
Rijstwafel (1 stuks)	❑	❑	❑	❑	❑	❑	❑	❑	❑
Franse kaas (ook op toastjes) (1 portie)	❑	❑	❑	❑	❑	❑	❑	❑	❑
Feta (4 blokjes)	❑	❑	❑	❑	❑	❑	❑	❑	❑
Salade (huzaren-, zalm-, kip-kerrie-, eier-, etc.)	❑	❑	❑	❑	❑	❑	❑	❑	❑
Tapenade, olijvenpasta (1 portie)	❑	❑	❑	❑	❑	❑	❑	❑	❑
Plakje worst. vleeswaren (ook op toastjes) (1 plakje)	❑	❑	❑	❑	❑	❑	❑	❑	❑
Augurk (1 stuks)	❑	❑	❑	❑	❑	❑	❑	❑	❑
Olijven (1 lepel)	❑	❑	❑	❑	❑	❑	❑	❑	❑
Broodje shoarma, worstbroodje, saucijzenbroodje (1 stuks)	❑	❑	❑	❑	❑	❑	❑	❑	❑
Loempia, bamibal, nassibal (1 stuks)	❑	❑	❑	❑	❑	❑	❑	❑	❑

Wilt u niet genoemde producten die u wel gebruikt, vermelden op bladzijde 26?

I. OVERIG

I1. Hoe vaak voegt u **zout, Aromat, Maggi of bouillon blokjes** toe tijdens de **bereiding** van uw voeding?

- ❑ Nooit
- ❑ Zelden
- ❑ Soms
- ❑ Meestal
- ❑ Altijd

I2. Hoe vaak voegt u aan **tafel zout, Aromat of Maggi direct** aan het eten toe?

- ❑ Nooit
- ❑ Zelden
- ❑ Soms
- ❑ Meestal
- ❑ Altijd

I3. Indien u regelmatig (ten minste wekelijks) **voedingsmiddelen en /of dranken gebruikt die niet op de lijst vermeld staan**, kunt u deze hieronder invullen.

Overige voedingsmiddelen	Hoe vaak per week	Hoeveel(-heid) per keer

I4. Bij sommige deelnemers hebben wij na de verwerking van de vragenlijst nog vragen. Mogen wii **contact met u opnemen** als wij naar aanleiding deze vragenlijst nog vragen hebben over uw voedingspatroon?

- ❑ Nee
- ❑ Ja

Deze vragenlijst is ingevuld op:

dag	maand	jaar

Einde vragenlijst. Bedankt voor uw medewerking !

U kunt de vragenlijst in de bijgeleverde envelop naar ons toesturen
