# Supplementary material for: Validation of a Semi-Quantitative Food-Frequency Questionnaire for Dutch Pregnant Women from the General Population Using the Method or Triads
Source: Nutrients. 2020 May 8;12(5):1341. doi: 10.3390/nu12051341 (PMC7284899; doi:10.3390/nu12051341)
Supplement: Supplementary file 1 [file nutrients-12-01341-s001.zip › Supplemental table S1 (Food groups).pdf]

Supplemental table S1

Food groups included in FFQ

-Potatoes and other tubers

-Vegetables

-Legumes

-Fruits

-Dairy products

-Cereals and Cereal products

-Meat and meat products

-Legumes

-Fish and shellfish

-Eggs and egg products

-Fat

-Sugar and confectionary

-Cakes

Non-alcoholic beverages

-Alcoholic beverages

-Condiments and sauces

-Soups, bouillon

-Miscellaneous
