# Supplementary material for: Validation of a Semi-Quantitative Food-Frequency Questionnaire for Dutch Pregnant Women from the General Population Using the Method or Triads
Source: Nutrients. 2020 May 8;12(5):1341. doi: 10.3390/nu12051341 (PMC7284899; doi:10.3390/nu12051341)
Supplement: Supplementary file 1 [file nutrients-12-01341-s001.zip › Supplemental table S2 (Correlations).pdf]

Table S2: Correlation coefficients between each of the three assessment methods and the validity coefficient calculated with the method of triads, adjusted for covariates [a]

				Fatty acids (plasma phospholipids)			
	Serum folate	RBC folate	Serum vitamin B_{12}	Saturated fatty acids	Monounsaturated fatty acids	Polyunsaturated fatty acids	Linoleic acid
	$n = 80$	$n = 77$	$n = 80$	$n = 78$	$n = 78$	$n = 78$	$n = 78$
Sample correlations							
r_{QM}	0.21	0.13	0.18	0.14	0.05	0.09	0.24
(95% CI)	(-0.04, 0.45)	(-0.13, 0.40)	(-0.09, 0.42)	(-0.10, 0.36)	(-0.17, 0.28)	(-0.14, 0.29)	(-0.03, 0,42)
r_{RM}	0.18	0.11	0.05	0.13	-0.04	0.02	0.20
(95% CI)	(-0.01, 0.39)	(-0.14, 0.39)	(-0.14, 0.25)	(-0.07, 0.33)	(-0.28, 0.19)	(-0.20, 0,22)	(-0.004, 0.40)
r_{QR}	0.43	0.41	0.24	0.42	0.24	0.41	0.36
(95% CI)	(0.22, 0.59)	(0.19, 0.58)	(0.07, 0.41)	(0.18, 0.62)	(-0.01, 0.46)	(0.17, 0.62)	(0.10, 0.59)
Validity coefficient [b]							
ρ_{QT}	0.71	0.70	0.91	0.64	NA	1.00	0.65
(95% CI)	(0.24, 1.00)	(0.18, 1.00)	(0.51, 1.00)	(0.16, 1.00)		(0.19, 1.00)	(0.23, 1.00)
Range [c]	0.21-0.71	0.13-0.70	0.18-0.91	0.14-0.64	NA	0.09-1.00	0.24-0.65

Abbreviations: FFQ, food-frequency questionnaire; NA, not available; rQM, correlation between FFQ and biomarker; rRM, correlation between 24h-recalls and biomarker; rQR, correlation between FFQ and 24h-recalls; rQT, validity coefficient of the FFQ; 95% CI, 95% confidence interval; RBC, red blood cell.

[a] *All correlations and coefficients are adjusted for BMI, smoking and gestational age at the time of blood sampling.*

[b] *Validity coefficients and confidence interval limits above 1 were set to 1.00.*

[c] *Range: The lower limit is rQM and the upper limit is ρ_{QT} (Ocke and Kaaks, 1997).*
