# Supplementary figures and images for: Validation of a Semi-Quantitative Food-Frequency Questionnaire for Dutch Pregnant Women from the General Population Using the Method or Triads
Source: Nutrients. 2020 May 8;12(5):1341. doi: 10.3390/nu12051341 (PMC7284899; doi:10.3390/nu12051341)

**Figure S3: Bland-Altman plots for energy-adjusted MUFA intake estimated by FFQ and 24h-recalls**

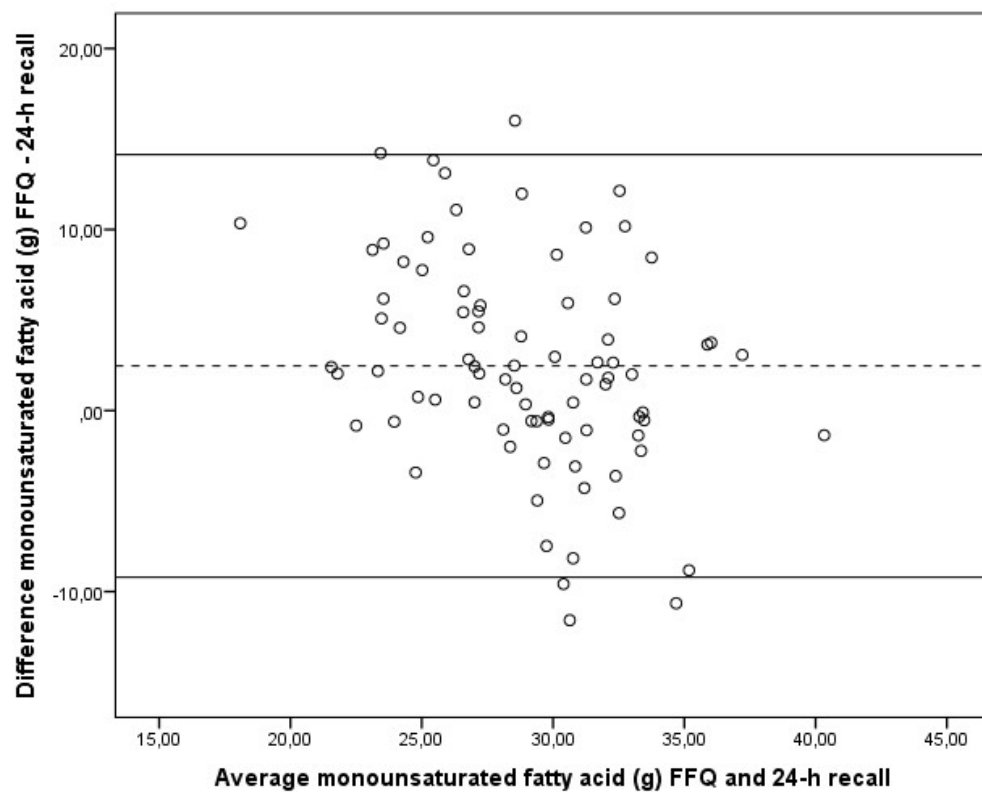

Supplement: Supplementary file 1 [file nutrients-12-01341-s001.zip › Supplemental Figure S3 (BA).pdf]

**Figure S4: Bland-Altman plots for energy-adjusted retinol intake estimated by FFQ and 24h-recalls**

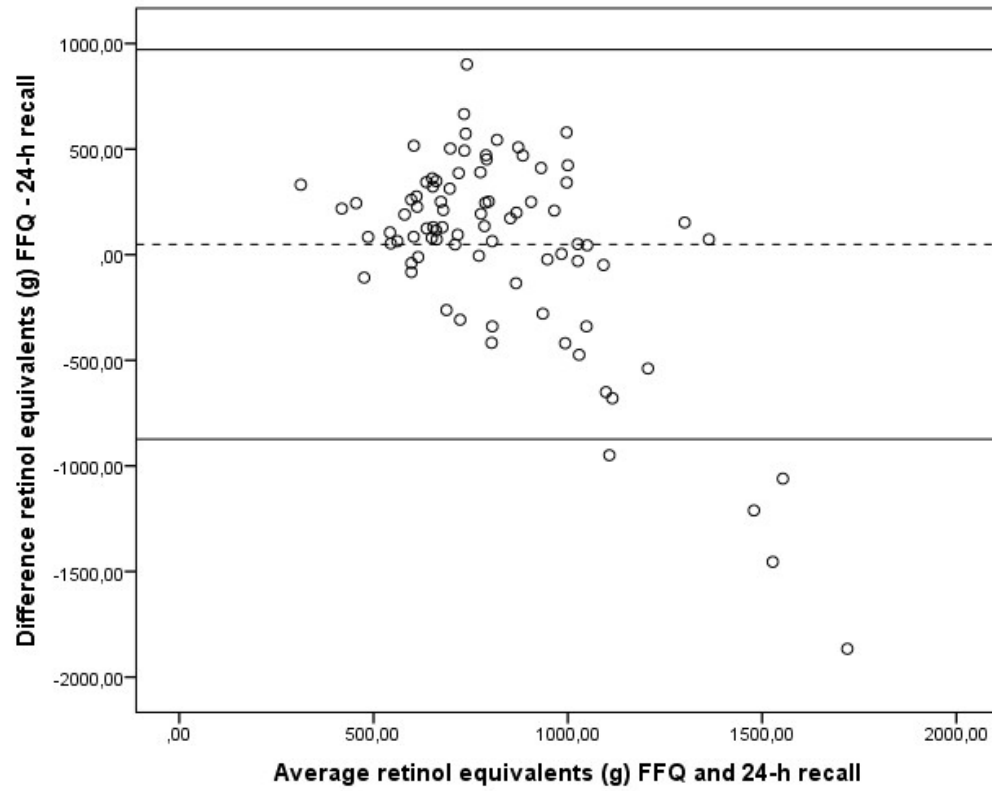

Supplement: Supplementary file 1 [file nutrients-12-01341-s001.zip › Supplemental Figure S4 (BA).pdf]

**Figure S5: Bland-Altman plots for energy-adjusted vitamin B1 intake estimated by FFQ and 24h-recalls**

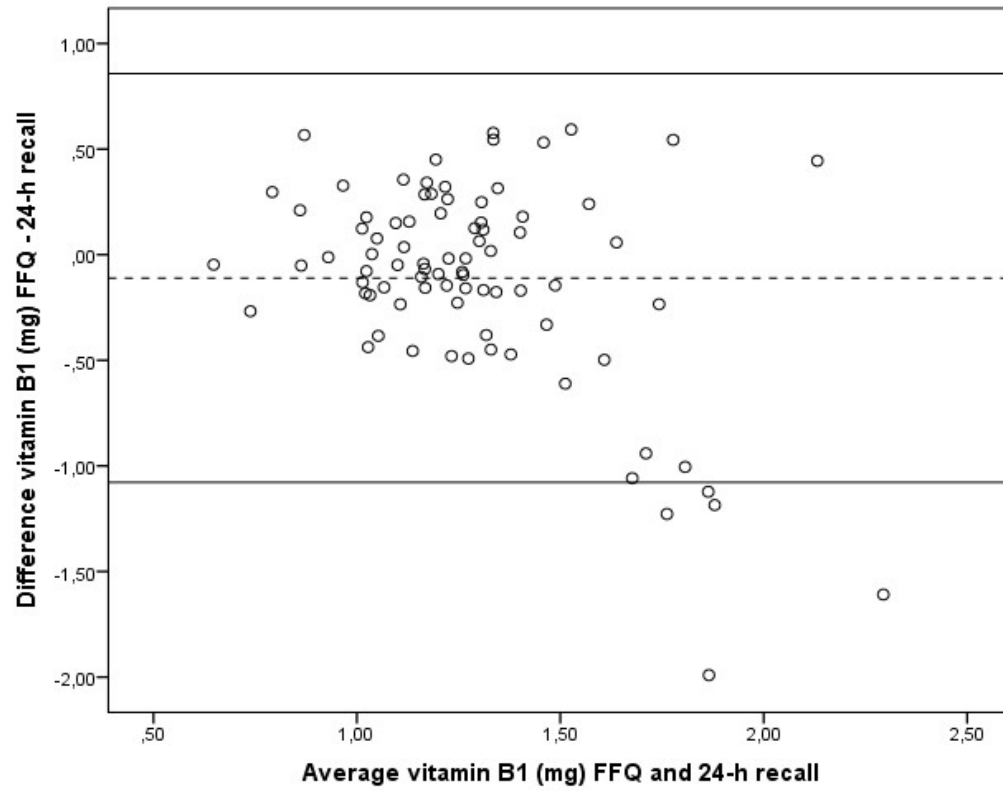

Supplement: Supplementary file 1 [file nutrients-12-01341-s001.zip › Supplemental Figure S5 (BA).pdf]

**Figure S6: Bland-Altman plots for energy-adjusted vitamin B12 intake estimated by FFQ and 24h-recalls**

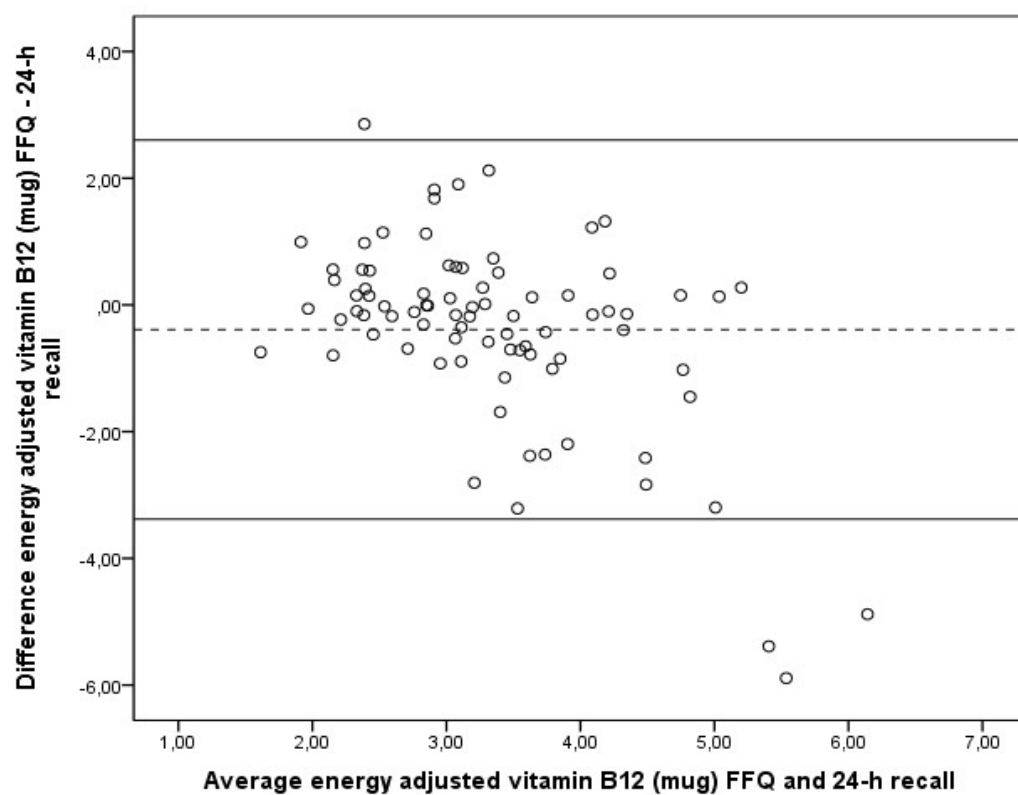

Supplement: Supplementary file 1 [file nutrients-12-01341-s001.zip › Supplemental Figure S6 (BA).pdf]

**Figure S7: Bland-Altman plots for energy-adjusted vitamin E intake estimated by FFQ and 24h-recalls**

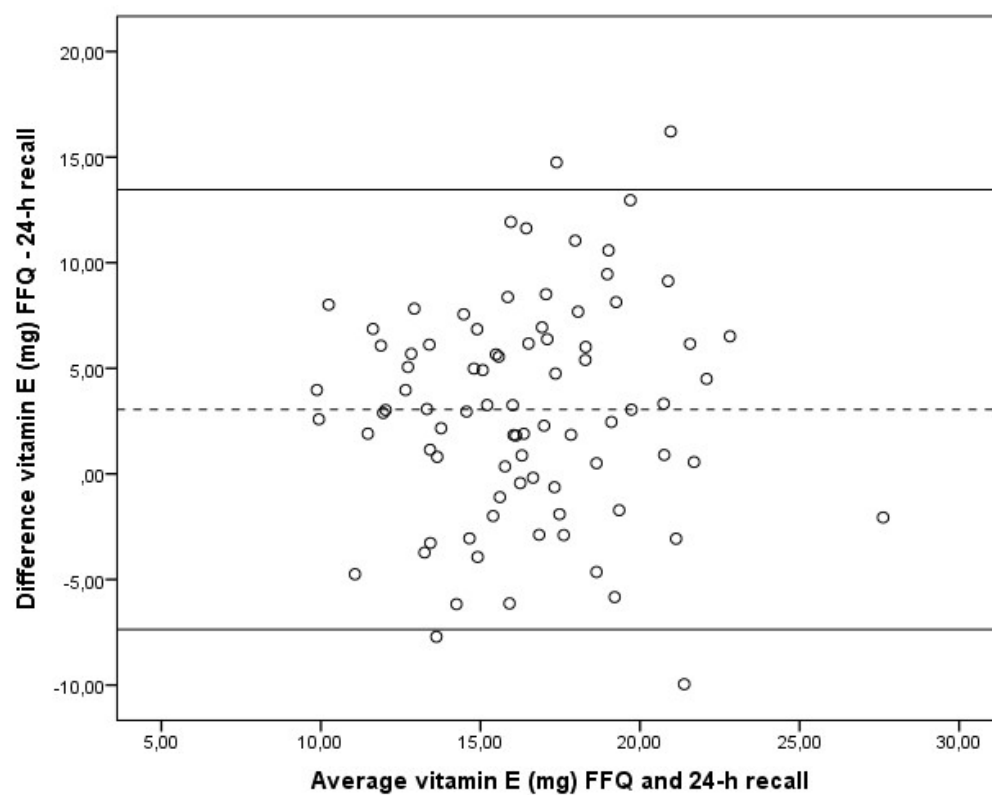

Supplement: Supplementary file 1 [file nutrients-12-01341-s001.zip › Supplemental Figure S7 (BA).pdf]
